# Dynamic Phytomeric Growth Contributes to Local Adaptation in Barley

**DOI:** 10.1093/molbev/msae011

**Published:** 2024-01-19

**Authors:** Yongyu Huang, Andreas Maurer, Ricardo F H Giehl, Shuangshuang Zhao, Guy Golan, Venkatasubbu Thirulogachandar, Guoliang Li, Yusheng Zhao, Corinna Trautewig, Axel Himmelbach, Andreas Börner, Murukarthick Jayakodi, Nils Stein, Martin Mascher, Klaus Pillen, Thorsten Schnurbusch

**Affiliations:** Leibniz Institute of Plant Genetics and Crop Plant Research (IPK), 06466 Seeland, Germany; Faculty of Natural Sciences III, Martin Luther University Halle-Wittenberg, Institute of Agricultural and Nutritional Sciences, 06120 Halle, Germany; Leibniz Institute of Plant Genetics and Crop Plant Research (IPK), 06466 Seeland, Germany; Leibniz Institute of Plant Genetics and Crop Plant Research (IPK), 06466 Seeland, Germany; Leibniz Institute of Plant Genetics and Crop Plant Research (IPK), 06466 Seeland, Germany; Leibniz Institute of Plant Genetics and Crop Plant Research (IPK), 06466 Seeland, Germany; Leibniz Institute of Plant Genetics and Crop Plant Research (IPK), 06466 Seeland, Germany; Leibniz Institute of Plant Genetics and Crop Plant Research (IPK), 06466 Seeland, Germany; Leibniz Institute of Plant Genetics and Crop Plant Research (IPK), 06466 Seeland, Germany; Leibniz Institute of Plant Genetics and Crop Plant Research (IPK), 06466 Seeland, Germany; Leibniz Institute of Plant Genetics and Crop Plant Research (IPK), 06466 Seeland, Germany; Leibniz Institute of Plant Genetics and Crop Plant Research (IPK), 06466 Seeland, Germany; Leibniz Institute of Plant Genetics and Crop Plant Research (IPK), 06466 Seeland, Germany; Center for Integrated Breeding Research (CiBreed), Georg-August-University, Göttingen, Germany; Leibniz Institute of Plant Genetics and Crop Plant Research (IPK), 06466 Seeland, Germany; German Centre for Integrative Biodiversity Research (iDiv) Halle-Jena-Leipzig, Leipzig, Germany; Faculty of Natural Sciences III, Martin Luther University Halle-Wittenberg, Institute of Agricultural and Nutritional Sciences, 06120 Halle, Germany; Leibniz Institute of Plant Genetics and Crop Plant Research (IPK), 06466 Seeland, Germany; Faculty of Natural Sciences III, Martin Luther University Halle-Wittenberg, Institute of Agricultural and Nutritional Sciences, 06120 Halle, Germany

**Keywords:** adaptation, architecture, barley, elongation, flowering-time genes, initiation phytomers

## Abstract

Vascular plants have segmented body axes with iterative nodes and internodes. Appropriate node initiation and internode elongation are fundamental to plant fitness and crop yield; however, how these events are spatiotemporally coordinated remains elusive. We show that in barley (*Hordeum vulgare* L.), selections during domestication have extended the apical meristematic phase to promote node initiation, but constrained subsequent internode elongation. In both vegetative and reproductive phases, internode elongation displays a dynamic proximal—distal gradient, and among subpopulations of domesticated barleys worldwide, node initiation and proximal internode elongation are associated with latitudinal and longitudinal gradients, respectively. Genetic and functional analyses suggest that, in addition to their converging roles in node initiation, flowering-time genes have been repurposed to specify the timing and duration of internode elongation. Our study provides an integrated view of barley node initiation and internode elongation and suggests that plant architecture should be recognized as a collection of dynamic phytomeric units in the context of crop adaptive evolution.

## Introduction

Plant architecture is the outcome of several successive developmental processes that can be classified into two sequential and coordinated morphogenetic events: organogenesis and extension (Barthélémy and Caraglio 2007). Organogenesis stems from the indeterminate, self-renewing meristems (stem cells) that give rise to different types of lateral organs (e.g. leaves and flowers) and axillary bud(s), plus the subtending internodes. These apex-derived organs form a functional unit called phytomer (Forster et al. 2007) that iterates and extends itself for several rounds until the apex either abscises/aborts (indeterminate growth) or terminates into a specialized structure (determinate growth). Consequently, a plant's architecture is a mosaic arrangement of different phytomers. Despite an individual's genetic uniformity, these mosaic phytomers can adopt various shapes in response to exogenous environmental constraints by, for example, altering meristematic determinacy or internode elongation (Harder et al. 2019; McKim 2020). Studying plant architecture is therefore of fundamental importance for understanding plant developmental biology and environmental adaptation.

**Fig. 1.**
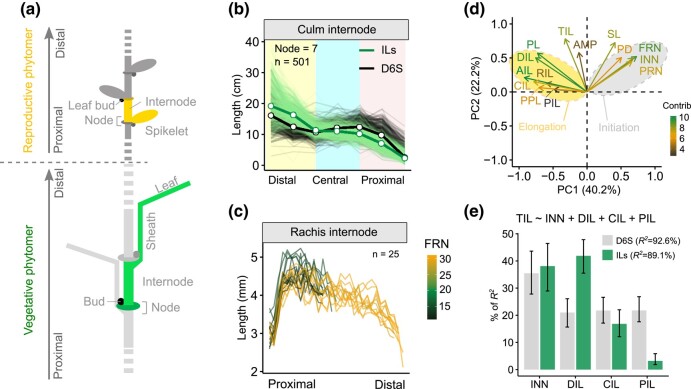
An oscillatory pattern of internode elongation and the node initiation—internode elongation relationship. **a.** Schematic representation of phytomeric structure from a barley vegetative culm and reproductive spike based on (Forster et al. 2007). A vegetative culm phytomer and a reproductive spikelet phytomer is highlighted with green and orange colors, respectively. Drawings are not to scale. **b.** Pattern of internode elongation from culms. Plants with a node number of 7 are shown. Bold lines are the average values. Distal, central and proximal internodes are highlighted with different colors according to supplementary fig. S4, Supplementary Material online. **c.** Pattern of internode elongation from spike rachis. FRN, final rachis node number. **d.** Loading plot of PC1 and PC2 from the 14 phenotypes representing node initiation and internode elongation. Note that initiation and elongation-related traits are largely separated based on the first two PCs. DIL, distal internode length; CIL, central internode length; PIL, proximal internode length; AIL, average internode length; PL, peduncle length; PPL, percentage of peduncle length; TIL, total culm internode length; AMP, culm length oscillatory amplitude; SL, spike length; PD, peduncle diameter; FRN, final rachis node number; INN, culm node number; PRN, potential rachis node number. **e**. Relative importance INN, DIL, CIL, and PIL to total culm length (TIL). A multiple linear regression analysis is formulated on the top of the bar plot. Upper and lower bound of the error bars are 95% confidence intervals based on 1,000 bootstrap replicates.

Barley (*Hordeum vulgare* L.) is a diploid inbreeding species considered a model of the *Triticeae* crops, which also include wheat (*Triticum* sp.) and rye (*Secale cereale* L.). Plant architecture of barley consists of two-ranked (distichous) arrangements of both leaves and floral organs (spikelets) on the alternate sides of vegetative culms and the reproductive axis (rachis), respectively. In this context, the main body axis of barley represents a simple and continuous segmentation of phytomers wherein both vegetative and reproductive organs co-exist at opposite ends (Fig. 1a). Barley is also an economically important crop that is usually grown at a high planting density where canopy shade is prevalent (e.g. ∼300 plants/m^2^ in Europe). Genetic modification aiming at improving barley plant architecture may therefore need to be implemented in the direction of high community uniformity with stable grain yield formation (Naylor et al. 1998). However, individual yield maximization may conflict with community performance (Weiner 2019; Abbai et al. 2020), especially for less lodging-tolerant crops such as barley (Dockter and Hansson 2015). A more fundamental understanding of how barley plant architecture is controlled is thus of high agricultural relevance.

Intrinsic and environmental response pathways that orchestrate plant architecture appear to act on several phytohormones, among which gibberellin (GA) plays a fundamental and essential role in cell elongation (Sachs 1965; McKim 2020). In particular, mutant alleles in the GA biosynthesis or signaling pathways have improved grain yield potential during the “Green Revolution” of wheat and rice (*Oryza sativa* L.) in the 1960s (Hedden 2003). Other phytohormones such as brassinosteroids (BRs), jasmonate (JA), and ethylene also modulate internode elongation via the GA pathway (Hattori et al. 2009; Dockter et al. 2014; Tong et al. 2014; Unterholzner et al. 2015; Patil et al. 2019). GA itself is not essential for organogenesis (Eshed and Lippman 2019); instead, florigen/antiflorigen, encoded by *FLOWERING LOCUS T* (*FT*)/*TERMINAL FLOWER 1* (*TFL1*) family genes, determines organ number by modifying the phase durations and expression of organ identity genes (Taoka et al. 2011). In barley, *HvFT1* integrates inputs from multiple signals for floral induction, such as photoperiod, vernalization, and the circadian clock. Key modulators for those input signals include *PHOTOPERIOD 1* (*PPD-H1*), *VERNALIZATION 1* & *2* (*VRN-H1*/*VRN-H2*), and *EARLY FLOWERING 3* (*HvELF3*) (Turner et al. 2005; Faure et al. 2012; Zakhrabekova et al. 2012; Boden et al. 2014; Deng et al. 2015). Mutations in any of these flowering-time genes frequently result in changes in both vegetative and reproductive phytomeric iterations (Huang et al. 2023), presumably via modulating meristematic determinacy (Melzer et al. 2008). In fact, floral induction is often coupled to an increase in GA concentration in the vegetative apex; consequently, many flowering plants elongate their internodes only after floral induction (King and Evans 2003), suggesting that the two hormonal systems (GA and [anti-] florigen) cooperate during morphogenesis. Nonetheless, how phytomer initiation and elongation are coordinated during morphogenesis remains poorly understood.

Here, we systemically investigated phytomer initiation and elongation by focusing on node number and internode length in the vegetative culms and reproductive spikes of barley (supplementary fig. S1, Supplementary Material online). We used a series of wild barley (*Hordeum vulgare* subsp. *spontaneum*) introgression lines (ILs) in the cv. Barke (an elite two-rowed cultivar) background (Maurer et al. 2015), as well as a diversity panel of domesticated barley (spring-type, six-rowed, hereafter D6S) representing the three major global-wide subpopulations relative to the center of barley origin (i.e. Eastern, Western and Ethiopian clades) (Milner et al. 2019) (supplementary table S1, Supplementary Material online). Our underlying assumption was that over thousands of years of barley evolution under domestication, the spread and fixation of beneficial alleles may have fine-tuned the plant architecture of barley to better adapt to local environments or high-density agricultural practices. Our results suggest that the barley plant (entity) is compartmentalized into dynamic phytomeric units, with distinct flowering-time genes functionally repurposed to determine the initiation and/or elongation of these units. We provide evidence that proximal internode elongation, a previously underexplored functional trait, is associated with both plant adaptation and reproductive efficiency (i.e. spikelet survival).

## Results

### A More Compact Inflorescence Architecture Developed Under Domestication

We primarily focused on reproductive traits in an initial survey of phenotypic diversity in the 25 wild barley founder parents of the HEB-25 population (Maurer et al. 2015) (supplementary fig. S2a-c, Supplementary Material online). We found that all inflorescence meristems from wild barley spikes were developmentally arrested and degenerated much earlier than those of cv. Barke; consequently, wild barleys had both lower potential rachis node number (PRN) at the maximum yield potential stage (Thirulogachandar and Schnurbusch 2021) and lower final rachis node number (FRN) at the anthesis stage. Notably, spike length (SL) was largely unchanged, consequently, wild barleys displayed longer rachis internode length (RIL) than cv. Barke. We conclude that evolution under domestication may have extended the apical meristematic phase to promote node initiation, but compromised subsequent internode elongation, resulting in a more compact inflorescence architecture. To further uncover the genetic underpinnings of these events, we selected four sub-families comprising 247 introgression lines (ILs) reflecting both the phenotypic and genotypic diversity of the wild barley gene pool (supplementary fig. S2d, Supplementary Material online).

### Internode Elongation Displays and Oscillatory Pattern

Barley culm internodes elongate successively in an acropetal direction, with the distal internodes (peduncle) being the shortest during spikelet differentiation stages, but becoming the longest after anthesis (Patil et al. 2019; McKim 2020). Our initial assumption was that without exogenous constraints, internode elongation would follow a linear pattern considering the continuity of indeterminate growth (Methods) (Barthélémy and Caraglio 2007). This means that the relationship of internode length to its position is to be adequately described by parametric linear regression. By measuring the culm internode length of ∼1,260 plants from the 247 ILs (∼5 replicates per genotype) at the anthesis stage, we determined that internode length showed an oscillating decline from the distal to proximal ends of the main axis. The overall pattern, however, did not follow a purely linear function, but rather displayed an “inverse-S’ curve that could be better explained by a nonlinear cubic function (Fig. 1b and supplementary fig. S3a-c, Supplementary Material online). Accordingly, culm internode elongation could be broadly trisected into distal, central, and proximal compartments. Further examination of ∼1,025 plants (∼4 replicates per genotype) from the D6S (domesticated six-rowed spring barley) panel demonstrated an overall conserved oscillatory pattern, but with distinct proximal–distal amplitudes intersecting at the central zone of the culm. The wild barley ILs tended to have longer internodes toward the distal end, and shorter internodes toward the proximal end compared with their D6S counterparts, and this pattern was independent of the total node number.

Interestingly, based on the measurement of 25 individual spikes from the wild barley ILs, we also observed a very dynamic elongation pattern for the rachis internodes, showing first oscillating increases and then decreases in internode length across the main rachis (Fig. 1c and supplementary fig. S4, Supplementary Material online). Collectively, our in-depth phenotypic observations uncovered a previously unrecognized oscillatory pattern for internode elongation and suggested that culm growth is compartmentalized.

### Phytomer Initiation and Elongation are Highly Related

We next examined the relationship between phytomer initiation and elongation using the phenotypic data mentioned above. The underlying assumption was that morphogenesis (e.g. the establishment of a phytomer) is composed of hierarchical and sequential developmental cascades, wherein functions of initiation genes can influence later differentiation and elongation (Waddington et al. 1983; Mizukami and Fischer 2000; Inzé and De Veylder 2006). In the barley inflorescence, initiation and its subsequent growth of the spikelet (rachis node) are molecularly decoupled due to pre-anthesis tip degeneration (Bonnett 1966; Huang et al. 2023; Shanmugaraj et al. 2023); we therefore used PRN as a quantitative readout of reproductive node initiation. We estimated the oscillation amplitude (AMP) for culm internodes by considering the deviation of the observed culm length from the expected linear pattern (Methods and supplementary fig. S3d and e, Supplementary Material online). Moreover, to better account for differences in node number among accessions, we standardized each accession by trisecting its culm while applying a moving average strategy to estimate the average lengths of distal (DIL), central (CIL), and proximal (PIL) internodes (Methods and supplementary fig. S5, Supplementary Material online). Other phenotypic details are summarized in supplementary fig. S1, Supplementary Material online. We sued 14 traits representing node initiation and internode elongation were used, which showed significant (*P* < 0.05) genetic variances with high repeatability ranging from 0.54 to 0.97 and broad phenotypic variation in both populations (supplementary fig. S6, Supplementary Material online, supplementary tables S1 to S3, Supplementary Material online).

Principal component analysis (PCA) in the ILs revealed that traits associated with node number and internode length could be largely separated by PC1 (40.2%), suggesting that plants with more nodes tended to have shorter internodes and *vice versa* (Fig. 1d). Indeed, a strong and negative correlation between node number and internode length was observed for both culms (*R^2^* = 0.34, *P* < 0.0001) and spikes (*R^2^* = 0.32, *P* < 0.0001). PCA also indicated a high correlation between the growth of vegetative and reproductive phytomers. For example, internode length (*R^2^* = 0.23, *P* = 9.4E-15) and node number (*R^2^* = 0.31, *P* < 0.0001) in the culm and the spike were positively correlated (supplementary fig. S7a-d, Supplementary Material online). AMP, together with several internode-length-related traits, further occupied the PC2 axis, which could explain 22.2% of the trait variations. A correlation analysis revealed that only distal internodes (i.e. peduncle and DIL), but not the central or proximal counterparts, were significantly correlated with AMP, indicating that a disproportional elongation of the peduncle might contribute to higher AMP. Indeed, we found that internode lengths were spatially correlated, and that elongation of distal internodes appeared to be largely independent from that of their proximal counterparts (supplementary fig. S7e, Supplementary Material online), revealing a high degree of independency for distal and proximal internode elongation. Finally, peduncle diameter (PD), the outcome of horizontal internode expansion, showed high positive loading on PC1 that was opposite from internode length, indicating opposing regulation of vertical and horizontal growth.

Because plant height (total culm length, or TIL) is determined by both node number (INN) and internode length, we assessed the relative contribution of INN and each length component (DIL, CIL, and PIL) to TIL (Fig. 1e). Using a multiple linear regression, we found that INN, DIL, CIL, and PIL together could explain ∼90% (*R*^2^ = 92.6% in the D6S and 89.1% in the ILs) of TIL variation. While the contribution of INN to TIL remained similar in both populations, the relative contribution of each length component varied: we observed a decline from 41.9% to 21.0% for DIL and an increase from 3.2% to 21.8% for PIL in the D6S versus the IL population. Considering the counteracting relationship between node initiation and internode elongation, this result provided evidence that phenotypic variation can be achieved in different phytomeric units without a major impact on the overall entity (herein plant height).

### Evolutionary and Genetic Basis of Node Initiation and Internode Elongation

**Fig. 2.**
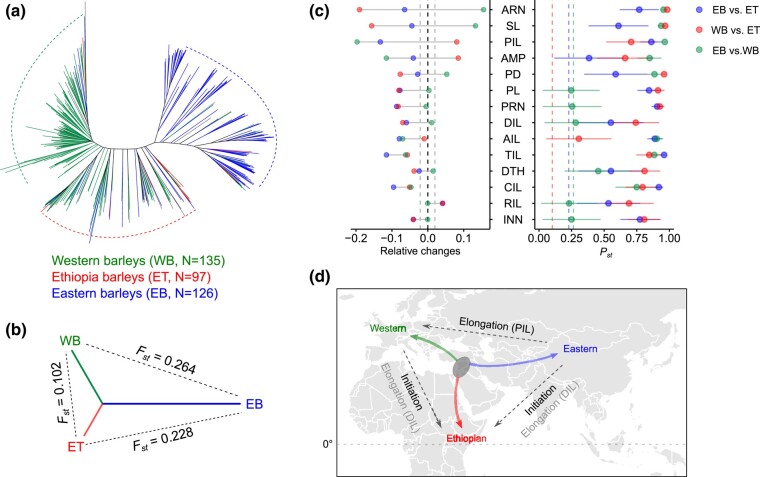
Evolution of phytomer initiation and elongation. **a.** An unrooted neighbor-joining tree clusters the 358 barleys (D6S) into three subpopulations corresponding to Eastern (green), Western (blue) and a mixed clade (red, Ethiopian clade). **b.** Pairwise comparisons of the genetic differentiation among the three subpopulations based on Fixation index (*F_ST_*). **c.** Pairwise comparisons of the phenotypic differentiation among the three subpopulations. Left panel shows the relative phenotypic changes for each subpopulation with respect to the whole population mean. Gray dashed lines mark the significant levels determined from two-tailed Student's *t*-test at *P* < 0.05. Right panel shows the phenotypic differentiation (*Q_ST_*) versus genetic differentiation (*F_ST_*). Colored dashed lines indicate the between populations mean *F_st_*. Error bars are 95% confidence intervals (CI_0.95_) for the phenotypic *Q_ST_* based on 1,000 permutations. EB, ET, and WB: Eastern, Ethiopian, and Western barleys. **d.** A simplified schematic diagram summarizing how phytomer initiation and elongation are changed during barley evolution based on the current phenotypic dissections. The gray oval highlights the proximate site of the Fertile Crescent where barley was first domesticated. We highlighted prominent phenotypic changes between subpopulations in black. Direction of the arrows points to the subpopulations with higher phenotypic values. Gray horizontal dashed line indicates the equator.

We sequenced (3- to 50-fold coverages) the genomes of the D6S population and obtained 22,405,297 bi-allelic Single-Nucleotide Polymorphisms (SNPs, minor allele frequency ≥5%), which then clustered the D6S population into the three well-defined subpopulations (Milner et al. 2019) (hereafter Eastern, Western and Ethiopian barleys [Methods and Fig. 2a and b]). A pairwise comparison of the phenotypes among the three subpopulations demonstrated an overall geographical relevance for the traits assayed (Fig. 2c and d). For example, a latitudinal cline of phytomeric initiation traits (INN and PRN) was observed from comparisons of Ethiopian barleys with the other two subpopulations, which was likely due to a slight delay in flowering time of Ethiopian barleys under greenhouse conditions. Furthermore, Ethiopian barleys had longer distal internodes (DIL and PL) and higher final spikelet number (FRN) compared with the other two subpopulations. By contrast, the trans-Eurasian comparison (Eastern vs. Western) revealed that days-to-heading (DTH), INN, PRN, as well as DIL and PL, were not significantly different between these subpopulations; instead, a major difference was observed for PIL, AMP, and PD. That said, compared with Western barleys, Eastern barleys had a “dwarf and sturdy” plant architecture owing to suppressed proximal internode elongation. Importantly, among the vegetative culm variables, PIL was negatively correlated (*r* = −0.31, *P* = 4.24 × 10^−7^) with spikelet survival, a key reproductive trait indicative of grain yielding efficiency (Kirby and Appleyard 1984; Kamal et al. 2022; Huang et al. 2023) (supplementary fig. S8, Supplementary Material online). Finally, using a *P_ST_*–*F_ST_* comparison strategy (Brommer 2011; Methods), we found that the observed morphological differences among different subpopulations were too large to be explained by random genetic drift (*P_ST_*>>*F_ST_*) (Fig. 2c, right), suggesting that selections drove the observed morphological divergences, and thus favored local adaptation.

**Fig. 3.**
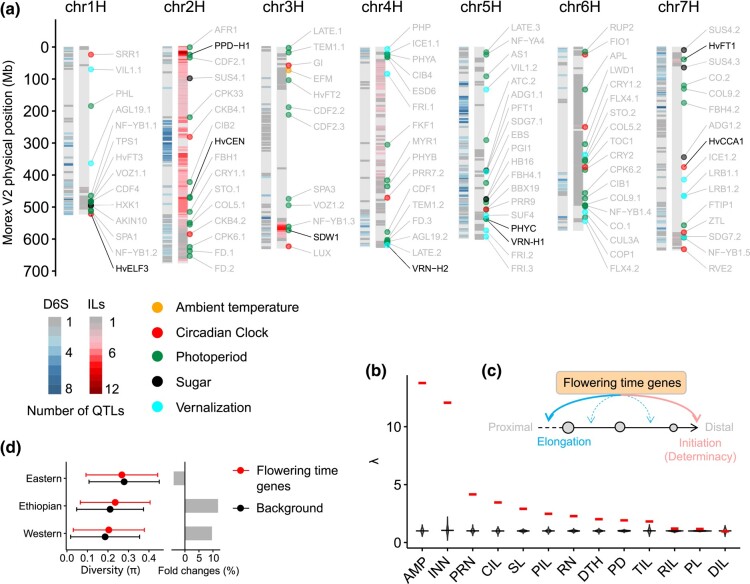
Genetic architecture of phytomer initiation and elongation. **a.** QTL density detected in D6S and ILs for the 14 traits. Barley homologs of *Arabidopsis* flowering-time genes from the FLOR-ID database are illustrated along the 7 barley chromosomes. To improve visualization, we only show genes interacting with environmental cues, or circadian clock (112 out of 268), and highlight those that have been experimentally validated in barley with black. **b.** SNP effects of flowering-time genes (268 in total) compared to the genome-wide random SNPs. Lambda (λ) values of SNPs within 200-kb of the 268 flowering-time genes (280,687 in total) are shown in red, violin plots are the distribution of λ values from 1,000 iterations of random SNPs. **c.** Schematic diagram depicting the functional repurposing of flowering-time genes in determining internode elongation based on the summary statistics in (**b**). Flowering-time genes are converged on node initiation (or meristematic determinacy (Melzer et al. 2008; Huang et al. 2023), indicated in pink curved arrow), and are repurposed to control internode elongation. Note that the unequal contributions of flowering-time genes to different internode elongation (e.g. PIL and CIL) may lead to a spatial unevenness of internode length, or higher amplitude (AMP), as observed in (**b**). **d.** Genetic diversity (π) of the 268 flowering-time genes in different subpopulations.

We next sought to understand the genetic basis of these 14 traits by performing a genome-wide association study (GWAS) (supplementary tables S4 to S6, Supplementary Material online). For the wild barley ILs, we detected 90 quantitative trait loci (QTLs, *P* ≤ 1e^−3^), each explaining 5.8% to 52.3% of the phenotypic variance, with wild barley alleles in general reducing node number but promoting internode elongation in the cv. Barke background (supplementary fig. S9, Supplementary Material online). For the D6S panel, we detected 30,363 marker–trait association events (*P* ≤ 1e^−5^), which were further clumped into 468 chromosomal regions ranging from ∼10-kb to ∼10-Mb with a median of ∼2.6-Mb encompassing 2,560 high-confidence genes. Plotting the QTLs from both populations (D6S and ILs) onto the barley chromosomes revealed a high degree of proximity (or overlap) in their genomic distributions, which were preferentially located toward the distal ends of each chromosome (Fig. 3a). Gene Ontology (GO) enrichment analysis of the 2,560 genes identified in the D6S population indicated that they were mainly involved in biological processes such as photoperiodism, response to cold, hypersensitive response, and GA homeostasis that are modulated by flowering-time genes (supplementary fig. S10, Supplementary Material online, supplementary table S7, Supplementary Material online). To interrogate this, we conducted a gene-based analysis by focusing on a list of putative flowering-time genes according to studies in *Arabidopsis* (268 in total, including several well-known genes highlighted in Fig. 3a, Methods). Results of a lambda (λ) analysis (Parvathaneni et al. 2020) showed that SNPs located within 200-kb of these genes tended to have a higher level of significance for the two initiation traits INN and PRN compared with genome-wide random SNPs (Fig. 3b), confirming the converging roles of flowering-time genes in meristematic determinacy and phytomeric iterations (Melzer et al. 2008; Huang et al. 2023). Interestingly, DTH per se was not the most significantly associated trait by the variations of flowering-time genes; instead, several elongation-related traits including AMP, CIL, and PIL tended to have greater reductions in *P*-values (Fig. 3b). Because PIL was insignificantly correlated with DTH (supplementary fig. S8b, Supplementary Material online, right), these results suggested that flowering-time genes could have broader biological relevance for phenotypic variations in internode elongation (e.g. PIL and CIL), which we propose as a functional repurposing (or adoption for a purpose other than DTH; Fig. 3c).

Finally, we found that Ethiopian barleys had a relatively higher genetic diversity (π) of flowering-time genes than the genome-wide background, whereas Eastern barleys showed the opposite trend (Fig. 3d). This confirmed the contribution of flowering-time gene variations in range-wide geographical adaptation (Russell et al. 2016) and suggested different selective strengths of these genes presumably due to contrasting environmental variables (i.e. photoperiod and temperature) across the broad geographical space.

In summary, our genetic analysis identified a priori candidates underpinning barley architectural adaptation. Below we detail three examples to demonstrate an uncoupled relationship for phytomer initiation and elongation: one for node initiation detected in the D6S population, one for internode elongation detected in both populations, and the last one for both initiation and elongation detected in the ILs.

### A Single Amino Acid Substitution in HvELF3 Promoted Node Initiation During the Northward Dispersal of Barley

**Fig. 4.**
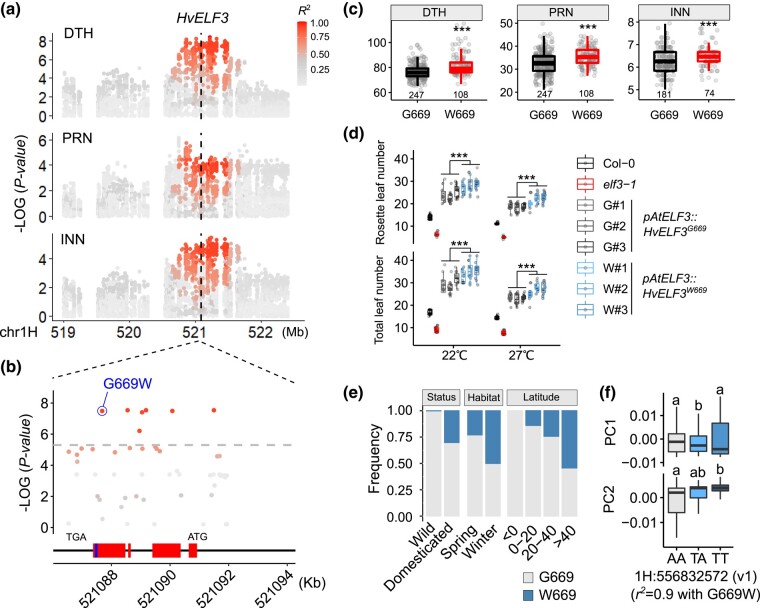
HvELF3 G669W variant contributes to more phytomeric iterations and northward expansion in domesticated barleys. **a–c.**  *HvELF3* natural variation is associated with DTH and phytomer initiation (PRN and INN). Local Manhattan plot (**a**) showing the association of the HvELF3 locus on DTH and initiation traits. Relationship (*R^2^*) of the peak SNP with the adjacent SNPs are shown. HvELF3 physical position is indicated with a dashed line. A non-synonymous SNP resulting in Glycine (G) to Tryptophan (W) substitution at position 669 of ELF3 protein is highly associated with the traits (**b**), with 669W showing delayed DTH and more phytomeric iterations (**c**). Red boxes indicate exons of *HvELF3*, and blue box indicates the putative PrD domain. **d.** Complementation test of HvELF3 variants (669G and 669W) in *Arabidopsis elf3-1* mutant at different temperatures. Three independent events (#1**–**#3) for each construct at T_2_ generation are used in the phenotypic comparison. **e.** Allelic frequency of HvELF3 G669W in different barley populations or geographical latitudes. We used the 300 re-sequenced barleys, including 100 wild barleys and 200 diverse domesticated barleys (Jayakodi et al. 2020), for the comparisons of domestication status (domesticated vs. wild) or growth habitat (spring vs. winter); we used capital latitudes from the D6S for the latitudinal comparison. **f.** Correlation of HvELF3 G669W variation with eigenvalues of the first two PCs (PC1 and PC2) representing the global domesticated barley diversity in the IPK Genebank collection (n = 19,778) (Milner et al. 2019). Note that the direct G669W SNP was not present in the Genotype-by-Sequencing (GBS) SNP matrix (Morex v1), we used the closely linked GBS SNP (1H:556832572, *r^2^* = 0.9) as a proxy of HvELF3 G669W. Significant levels in **c** and **d** are determined from two-tailed Student's *t*-test. ***P < 0.001. *n* = 11 or 12 replicates in **d**. Letters above the boxplot in (**f**) represent statistical significance from ANOVA followed by Tukey's HSD test (*P* < 0.01).

One locus on chr1H detected in the D6S panel was found to be highly associated with PRN (*P* = 6.61 × 10^−7^) and DTH (*P* = 4.03 × 10^−9^), marginally associated with INN (*P* = 5.98 × 10^−6^), but insignificantly associated with elongation-related traits (Fig. 4a-c). Plants with the minor allele (see below) at this locus were taller than those with the major allele owing to a greater INN (supplementary fig. S11a, Supplementary Material online). This locus contained the barley *EARLY FLOWERING3* (*HvELF3*) gene, which encodes a component of the circadian clock acting as a DTH repressor (Faure et al. 2012; Zakhrabekova et al. 2012; Boden et al. 2014). Previous studies indicate that *HvELF3* reduced-function mutations accelerate reproductive transition and facilitate short-season adaptation in two-rowed spring barleys (Faure et al. 2012; Zakhrabekova et al. 2012). However, analysis of sequence variation at *HvELF3* in the D6S population revealed that the GWAS hit was not caused by any of the induced mutations reported previously. Instead, we identified a non-synonymous SNP among the top SNPs, resulting in an amino acid substitution from Glycine (G) to Tryptophan (W) at position 669 close to the C-terminus (here after G669W; Fig. 4b). This substitution appeared to be located in a putative prion-like domain (PrD) previously determined to be essential for thermal responsiveness in *Arabidopsis*, with ELF3 variants lacking the PrD domain showing constitutive repression of flowering (Jung et al. 2020). Our *in silico* prediction (Lancaster et al. 2014) indicated that W669-type HvELF3 proteins have a narrower PrD domain than the G669-type (supplementary fig. S11b, Supplementary Material online); in accordance with this, W669-type plants showed delayed DTH and greater PRN and INN compared with plants with the G669-type HvELF3 (Fig. 4c), suggesting that W669 might be a gain-of-function mutation. To further test this, we fused the *Arabidopsis ELF3* promoter sequence (3542-bp) with coding sequences from both HvELF3^G669^ and HvELF3^W669^ to generate two transgenic constructs (*pAtELF3::HvELF3^G669^* and *pAtELF3::HvELF3^W669^*). We introduced these into the *Arabidopsis elf3-1* mutant (Jung et al. 2020) and obtained three independent transformation events (#1–#3) for each construct. We found that both constructs could complement the *elf3-1* mutant phenotypes well in terms of leaf number and flowering time at either 22 °C or 27 °C. Importantly, plants transformed with HvELF3^W669^ tended to have more leaves and delayed flowering compared with plants transformed with HvELF3^G669^ at both temperatures (*t-test*, *P* < 0.001) (Fig. 4d and supplementary fig. S11c, Supplementary Material online), supporting G669W as a functional variant.

The G-variant of ELF3 appeared to be restricted to plant species inhabiting colder climates (supplementary fig. S11b, Supplementary Material online). Importantly, we discovered that the W669 allele was almost absent from wild barleys (1 of 100) (Jayakodi et al. 2020), but has emerged at higher frequencies with increasing latitude (northward expansion), in particular, in winter barleys (Fig. 4e), suggesting that W669, which delays reproductive transition, may facilitate winter survival during northward expansion. Supporting this, wild barley ELF3 alleles (G669-type) accelerate plant development (Zhu et al. 2022; Zahn et al. 2023). We extended our analysis to the whole IPK barley Genebank, whose diversity space could be best represented by the first two axes of a PCA corresponding to longitudinal (PC1: Eastern vs. Western) and latitudinal (PC2: Ethiopian vs. others) gradients, respectively (Milner et al. 2019). The G669W variant was highly correlated with the PC2 eigenvalue, but not that of PC1 (Fig. 4f and supplementary fig. S11d, Supplementary Material online), reinforcing an important role for the HvELF3 G669W variation in latitudinal adaptation. Collectively, our results indicate that HvELF3 G669W extended the barley phenology (seasonal timing of the lifecycle) to facilitate more phytomeric iterations and suggest an uncoupled regulation for internode elongation and node initiation/flowering time.

### Divergent Haplotypes of a Superlocus Have Cumulatively Compacted Plant Architecture During the Eastward Dispersal of Barley

**Fig. 5.**
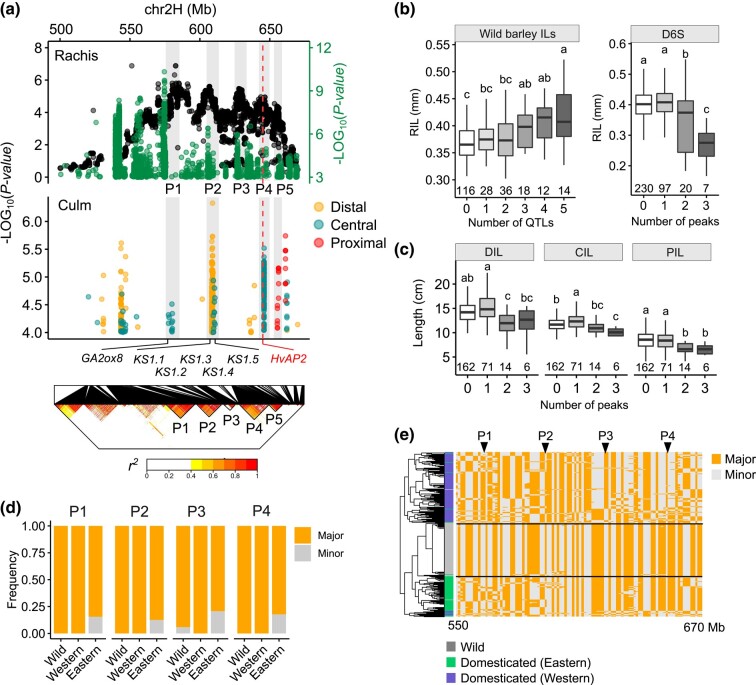
Identification of a superlocus associated with internode length. **a.** Dotplot showing the association signals for internode length. Black and green dots from the top panel are association signals detected in the wild barley ILs and the D6S population, respectively. Five adjacent peaks (P1**–**P5) supported by both populations are highlighted with gray shadows. *HvAP2* position is marked with a red dashed line. Locations of prime candidates involved in GA metabolism are indicated. Bottom panel shows a local linkage disequilibrium (LD) heatmap based on 1,034,167 SNPs (500 to 670-Mb) from the D6S population. **b, c**. Boxplots showing the effects of P1**–**P5 on rachis internode length (RIL) (**b**) and culm internodes (**c**). Wild barley alleles additively increase RIL, whereas domesticated alleles additively decrease RIL, as well as culm internodes (DIL, CIL, and PIL). Note that no domesticated barleys with stacking of up to four or five peaks can be identified in the D6S population. Letters above boxplot represent statistical significance from one-way analysis of variance (ANOVA) followed by Tukey–Kramer honestly significant difference (HSD) tests. **d.** Allelic frequencies based on the GWAS peak SNPs from each of the four peaks (P1**–**P4) in wild, Eastern, and Western barleys. SNP data are extracted from a previous study (Jayakodi et al. 2020). **e.** Haplotype diversity within the superlocus in wild, Eastern, and Western barley populations. Barley accessions are hierarchically clustered according to 170 binned polymorphic sites from 1,274,896 SNPs (Methods). Locations of P1**–**P4 are indicated.

We next focused on a large genomic segment encompassing ∼100-Mb on the long-arm of chromosomal 2H (Morex V2: 550 to 650-Mb) that was mainly associated with internode elongation, such as rachis (in both populations) and culm (in D6S) (Fig. 5a, supplementary fig. S12 to S14, Supplementary Material online). Multiple physically close peaks (P1–P5) appeared in this large segment, and, notably, peaks for culm DIL, CIL, and PIL recapitulated their spatial relationships. For example, shared peaks were detected for DIL and CIL (P2) and for CIL and PIL (P5), but not for DIL and PIL. In addition, we also detected both PIL- and CIL-specific peaks; the latter was co-localized with *HvAPETALA2* (*HvAP2*), a gene known for controlling both rachis and culm internode length (Houston et al. 2013; Patil et al. 2019), and coincided with the P4 region detected in the ILs. A previous study suggested that variations in the *microRNA172* (*miR172*) binding site of *HvAP2* result in lower *HvAP2* expression and shorter internode length. However, we did not find any sequence variations within the *miR172* region of the founder parents of the wild barley ILs, nor were any of the GWAS peaks tagged by the *miR172* variations, suggesting that mutations outside of the *HvAP2 miR172* binding site may be responsible for P4 (supplementary fig. S14, Supplementary Material online).

We detected at least eight independent linkage disequilibrium (LD) blocks (*r^2^* ≥ 0.4) within the region encompassing the five shared peaks (P1–P5) in the D6S population (Fig. 5a, bottom). Importantly, we were able to identify diverse allelic combinations in both the wild barley ILs and the D6S accessions that, when more alleles were stacked, showed an additive increase in rachis internode length (RIL) in the wild barley ILs but an additive decrease in internode length in the D6S population (Fig. 5b and c); the additive effect was not observed for initiation traits (supplementary fig. S12, Supplementary Material online). This indicated that multiple independent causal genes for internode elongation were present within the segment and that domesticated and wild barley alleles likely have a different functional status. Consistent with this, analysis of allele frequency based on the GWAS peak SNPs in each of the five blocks showed that the minor alleles were almost exclusively from wild barleys and were preferentially distributed in Eastern barleys (Fig. 5d). We observed a relatively high haplotype diversity across this interval in the domesticated barleys compared with the wild barleys (Fig. 5e), making it difficult to exclude any significant SNPs as tags of other contributing variants. Thus, we postulate that this ∼100-Mb genomic segment is a superlocus that contains multiple independent functional haplotypes (including *HvAP2*) contributing to a mosaic of introgression blocks, which have cumulatively compacted the plant architecture of Eastern domesticated barleys.

Because GA is known to determine internode elongation in a dosage dependent manner, which would fit the additive mode of action observed above (Boden et al. 2014; Youssef et al. 2017), we searched for genes related to GA metabolisms within the interval as a priori candidates. Within P1–P5, P1, and P2 appeared to be more relevant in achieving phenotypic changes compared with the others (supplementary fig. S13, Supplementary Material online). Of particular note, we identified at least six candidates (Fig. 5a), including one encoding for a GA2ox8 homolog in the P1 region, and five for *ent*-kaurene synthases (KSs) participating in the GA biosynthetic pathway (Sun and Kamiya 1994) in the P2 region. All of these candidate genes carried either non-synonymous or nonsense mutations specific to Eastern barleys (supplementary fig. S14, Supplementary Material online). Taken together, our genetic studies revealed that internode elongation is largely regulated by additive genetic pathways, presumably via modulating GA homeostasis; they further showcased multi-functional haplotypes in one superlocus contributing cumulatively to quantitative trait variation, which may have implications for other GWAS and causal-gene studies.

### The PPD-H1–*SDW1* Regulatory Module Coordinates Node Initiation and Internode Elongation

**Fig. 6.**
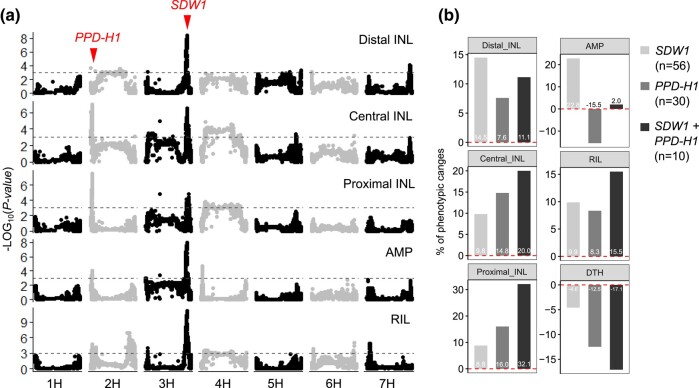
*PPD-H1* and *SDW1* loci cumulatively contribute to internode elongation. **a.** Manhattan plots showing the associations for five internode elongation-related traits. Locations for *PPD-H1* and *SDW1* are highlighted. Gray dashed lines are genome-wide threshold at *P* = 0.001. **b**. Allelic stacking shows the additive or synergistic effects for *PPD-H1* and *SDW1* on internode elongation, as well as flowering-time phenotypes collected previously (Maurer et al. 2015). Mean phenotypic values from each combination are normalized according to those without wild barley alleles at both loci (*n* = 120).

Our last example focuses on two loci with large effects detected in the wild barley ILs: one for both node initiation and internode elongation that coincided with the *PPD-H1* gene on chr2H; the other specifically for internode length that coincided with *SDW1* encoding a GA20ox2 involved in gibberellin (GA) biosynthesis on chr3H (Fig. 6a). When using the above-mentioned PCA loadings (PC1 and PC2) as trait variables, these two loci together explained 71.9% of PC1 variation and 54% of PC2 variation (supplementary fig. S15a, Supplementary Material online). Both *PPD-H1* and *SDW1* are believed to be nonfunctional (or show reduced functionality) in cv. Barke but fully functional in all four wild barleys, according to functional mutations reported previously (Turner et al. 2005; Xu et al. 2017; Sharma et al. 2020). Intriguingly, we observed a cumulative shift of the main effects for the two loci on spatial culm internode elongation (Fig. 6a). For example, wild barley *SDW1* alleles had a major effect on DIL, but *PPD-H1* became the dominant locus for PIL variation. Importantly, both loci showed a synergistic epistatic interaction for PIL and additive effects for RIL and DTH, as reported previously (Fig. 6b) (Maurer et al. 2015). The spatial effects on internode elongation for these two loci also applied to the rachises, as functional alleles at both loci promoted longer central-proximal rachis internodes (supplementary fig. S15b, Supplementary Material online).

**Fig. 7.**
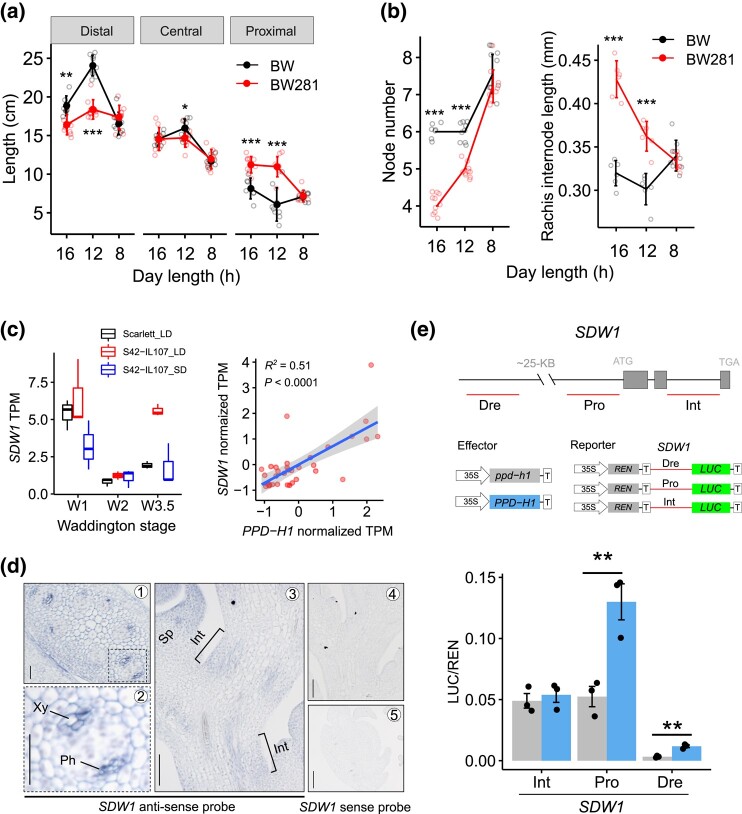
*PPD-H1* is molecularly coupled with *SDW1*. **a.** Quantitative comparison of distal, central and proximal internode length under 16, 12, or 8 h (h) of day-length conditions. **b.** Quantitative comparison of culm node number (left panel) and average rachis internode length (right panel) under 16, 12, or 8 h (h) of day-length conditions. **c.** PPD-H1 positively regulates *SDW1* gene expression. Boxplot shows the *SDW1* transcripts in developing apexes of NIL-*PPD-H1* (S42-IL107) and the control (Scarlett) under short-day (SD) or long-day (LD) conditions. Dotplot shows the co-expression of *SDW1* and *PPD-H1* across diverse tissue types and developmental stages. RNA-Seq data reported previously (Digel et al. 2015; Thiel et al. 2021) are used to estimate the transcripts (TPM) based on Morex annotation v2. **d**. *SDW1* mRNA *in situ* hybridization in developing rachis during the elongation stage (W5.5) of spike development. Transverse (**d1**, **d2**, and **d5**) or longitudinal (**d3** and **d4**) sections hybridized with an antisense probe (**d1–d3**) or a sense probe (**d4** and **d5**, control) of *SDW1* are shown. Xy, xylem; Ph, phloem; Int, rachis internode; Sp, spikelet. Scale bars: 20 μm in **b1** and b2; 50 μm in **d3–d5**. **e.** Dual-LUC assay showing the direct activation of *SDW1* by PPD-H1. The diagram on the top depicts the regions used for the assay, including a distal regulatory region (Dre), the 2-kb promoter region (Pro), and the second intron (Int) of *SDW1*. ATAC-seq data reported previously (Lu et al. 2019) are used to estimate the accessible chromatin regions of *SDW1*. Relative luciferase activities (LUC/REN) are determined in barley leaf protoplasts co-transfected with different effector and reporter constructs. Data are shown as mean ± s.d. (*n* = 3 biological replicates). Significant levels in (**a**), (**b**), and (**e**) are determined from two-tailed Student's *t*-test. *P < 0.05; **P < 0.01; ***P < 0.001. *n* = 3 to 9 replicates.

Our genetic analysis also revealed that both *PPD-H1* and *SDW1* loci were significantly associated with AMP, but with opposite effects. Consequently, stacking both loci offset their individual effects on AMP (Fig. 6b). Because distal and proximal internode elongations were oppositely associated with AMP during flowering (supplementary fig. S7e, Supplementary Material online), we hypothesized that proximal internode elongation modulated by PPD-H1 may balance the distal counterparts, resulting in a plant architecture with more evenly spaced nodes and internodes. To further investigate this, we compared the phenotypes of a *PPD-H1* near-isogenic line (BW281; carrying the photoperiod sensitive, functional *PPD-H1* allele) and the wild-type control (Bowman, hereafter BW). Under long-day conditions (16 h of light), plants and spikes of BW281 were typically shorter, mainly resulting from a severe reduction in phytomer number. However, measurements for each of the internodes demonstrated that internode length was increased in BW281, in particular at the proximal ends. Importantly, differences for node initiation and internode elongation among BW281 and BW became insignificant as day length became shorter (Fig. 7a and b and supplementary fig. S15c, Supplementary Material online), suggesting that functional PPD-H1 (BW281) integrates photoperiod signals to coordinate both node initiation and internode elongation (in particular, the proximal internodes).

The additive or synergistic (i.e. for proximal internodes) genetic relationship between *PPD-H1* and *SDW1* suggests a possible molecular interaction of these genes for internode elongation. Indeed, analysis of transcriptomic data generated from developing spikes of cv. Scarlett and S42-IL107 (an introgression line in the Scarlett background that carries the photoperiod sensitive *PPD-H1* allele from wild barley) (Digel et al. 2015) revealed that PPD-H1 positively regulates *SDW1* gene expression in a photoperiod-dependent manner (Fig. 7c, left); likewise, S42-IL107 produced longer rachis internodes than Scarlett (supplementary fig. S15b, Supplementary Material online). Moreover, we found that *SDW1* was highly co-expressed with *PPD-H1* in several diverse tissue types comprising a broad range of spike developmental stages (Thiel et al. 2021) (Fig. 7c and supplementary fig. S16a, Supplementary Material online). Interestingly, *SDW1* was more highly expressed in the central sections of developing spikes in both BW and *tip sterile 2.b* (*tst2.b*), a mutant displaying premature rachis internode elongation (Huang et al. 2023). A similar gene expression pattern for *PPD-H1*, however, was only observed in *tst2.b*, which was consistent with the rachis internode elongation patterns along the spike (supplementary fig. S16b and c, Supplementary Material online). We previously showed that *PPD-H1* mRNA was detected in the inflorescence vasculature during spike development (Huang et al. 2023). Importantly, in situ *SDW1* mRNA signals were also detected in inflorescence vasculatures and rachis internodes (Fig. 7d), consistent with its function in rachis elongation. We examined whether PPD-H1 may regulate *SDW1* expression via interacting with regulatory regions close to *SDW1*. Analysis of chromatin accessibility in *SDW1* revealed several accessible regions that could potentially be binding targets of upstream transcription factors (supplementary fig. S17, Supplementary Material online). In a dual-luciferase (LUC) assay, we observed approximately twofold greater LUC activity from co-incubations of PPD-H1 with the distal region and the promoter than from co-incubations of ppd-H1 with these regions, although the distal region was less transcriptionally active than the promoter (Fig. 7e), indicating transcriptional activation of *SDW1* by PPD-H1 during internode elongation.

Altogether, these results demonstrate that the phytomer initiation gene *PPD-H1* is repurposed to regulate internode elongation via the GA pathway acting through the vasculature. This may have broad implications in plant architectural redesign for proximal culm internodes and spike compactness.

## Discussion

In this study, we systematically investigated phytomer initiation and elongation patterns and their genetic underpinnings, and made three fundamental discoveries:

First, we found that a general tendency of barley domestication is the compactness of the inflorescence architecture, which was due to both an extension of phytomeric iterations (indeterminacy) and subsequently the restriction of internode elongation (supplementary fig. S2, Supplementary Material online). These observations may add insights into the evolution of spike-type inflorescence under domestication, which has been found to be morphologically more similar to their wild progenitor with respect to that of rice or maize (Harlan and Zohary 1966; Haas et al. 2019).

Second, we showed that phytomer elongation is an oscillatory process and is spatially partitioned into distal, central, and proximal compartments. This represents a marked advance from previous work because we were able to define an underexplored, functional, and supporting feature of internode elongation beneath the canopy, whose genetic underpinnings and biological implications are different from those of their distal counterparts (i.e. peduncle). Importantly, proximal internode growth has undergone directional selection during barley adaptation. We postulate that a similar lengthening rule may apply to other cereal crops, such as wheat and rye, considering the overall conserved phenology of these species (McMaster 2005).

Third, our genetic analysis points to a functional repurposing of flowering-time genes for internode elongation. This is because the architecture of all cereal crops, including barley studied here, are segmented into functional phytomeric units. That current floral induction model of mobile signals from leaf to shoot apex is not sufficient to explain the dynamic patterns of different phytomeric elongations, which usually take place after the floral induction. In fact, throughout the plant lifecycle, many traits such as seed size are photoperiodically controlled via flowering-time genes without having direct contact with the floral induction window (Gendron and Staiger 2023; Yu et al. 2023). Our current genetic studies may thus allow a deeper understanding of the dynamic growth strategies of cereal crops.

### Proximal Internodes as an Underexplored Functional Trait

Architecturally, height increase may confer fitness advantages under natural conditions, such as benefiting pollination and allowing plants to outcompete neighbors for light, and is largely achieved through the flowering-induced elongation of distal internodes (i.e. peduncle) (McKim 2020). However, under a monoculture farming environment, plant cultivation is usually driven toward community uniformity and stable yield formation (Abbai et al. 2020). This requires plants to stabilize their growth in response to a heterogeneous environment at different growth stages. For example, during the pre-anthesis spikelet initiation/differentiation stages, stem elongation usually starts at the proximal internodes instead of the distal counterparts. During this particular developmental window, proximal internodes are spatiotemporally proximate to many unique stresses beneath the canopy layer, such as light (discussed below), temperature, and moisture. In this context, appropriate switching down of the environmental response machinery, thereby dampening plants’ growth plasticity, may benefit community performance. In particular, shorter proximal internodes are associated with higher spikelet survival (supplementary fig. S8, Supplementary Material online) because longer internodes would mean extra inputs (McKim 2020). It can therefore be speculated that shortening of the proximal internodes enables greater resource reallocation to the developing spikes, thereby improving spikelet survival. Optimal proximal internode elongation is also directly relevant to lodging because bending and breaking of stems usually take place near the ground, making longer internodes unfavorable for lodging resistance. Lodging remains a more common problem in barley than in rice and wheat cultivars (Dockter and Hansson 2015). Our work may therefore provide a conceptual framework for enhancing the genetic gains of proximal internode growth for a sustainable grain yield.

### Light Regime as a Driving Force for Dynamic Vertical Growth

The vertical growth portioning of the internodes immediately suggests light regimes (e.g. red/far-red, R/FR, and light ratio) as possible driving forces for selection considering the light gradient at different canopy layers (Davis and Simmons 1994). This is aligned with recent findings demonstrating that increasing canopy light transmission can shorten the proximal–central internodes in both rice and wheat (Zhong et al. 2020; Golan et al. 2022). Indeed, a vertical light gradient has already been established at the tillering/elongation stages and becomes more evident at the anthesis stage under greenhouse conditions (supplementary fig. S18, Supplementary Material online). In fact, barley *PHYTOCHROME* (*PhyA*–*C*) homologs were amongst the candidates for different internode elongations in the present study (Fig. 3, i.e. *PhyA* for AMP on 4HS; *PhyB* for CIL/PIL on 4HL; and *PhyC* for CIL on 5HL). Other photoreceptor genes, including *CRYPTOCHROME1* (*CRY1*), were also found to be within the quantitative trait locus (QTL) region for DIL and PL on 2HL. Light perceived by these photoreceptors induces a series of physiological changes, e.g. endogenous clock synchronization and photoperiod responses (Jiao et al. 2007). One scenario is that different internodes may have to synchronize their own clock period to match the vertical dynamic light gradient, resulting in the oscillatory elongation of internodes. This is supported by a recent finding that field microenvironments with different degrees of self-shading lead to adjustment of the endogenous clock in different leaves, thereby impacting agronomic performance (Dantas et al. 2021). In this context, the shorter proximal internodes observed in Eastern barleys (many are naked barleys from the Tibetan Plateau) could represent a local adaptation to high solar radiation such as that seen in the Tibetan Plateau (Norsang et al. 2009), during which the photoperiodic response machinery must be switched down (Liu et al. 2017). Consistent with this view, our data also suggest that photoperiod insensitivity is coupled with shorter proximal culm internodes. The fact that flowering time between Eastern and Western barleys showed no significant difference under our greenhouse conditions (long-day) further suggests that the photoperiodic control of internode elongation can be uncoupled from flowering-time variation, which is in agreement with recent research in rice demonstrating that photoperiodic response and GA-dependent internode elongation can be uncoupled (Gómez-Ariza et al. 2019). In this scenario, the barley flowering-time gene *PPD-H1* is repurposed to determine proximal internode elongation via (in part) *SDW1*-mediated GA pathway.

### Balancing the Phytomer Initiation and Elongation Tradeoff to Optimize Height

**Fig. 8.**
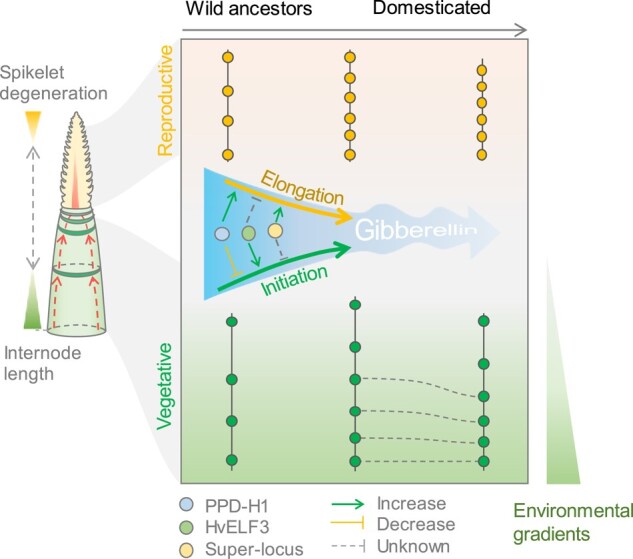
A proposed model depicting how phytomer initiation and elongation are changed under domestication or crop adaptation. During the spikelet initiation/differentiation phase, proximal internode length is positively associated with tip spikelet degeneration, presumably due to extra inputs for elongations or transports (indicated with red dashed arrows). Wild barleys tend to have longer proximal internodes and early tip degeneration. The transition from this “initiated less, elongated more” of wild ancestor to the “initiated more, elongated less’ of domesticated barley can be due to genetic reshufflings of flowering-time genes that result in a dynamic but overall reduction of GA levels, as demonstrated here three examples: the PPD-H1—*SDW1* axis, the superlocus, and the HvELF3 (Boden et al. 2014). The most prominent changes toward the “semi-dwarf” stature of domesticated barleys in Eastern barleys are the shortening of proximal internodes (indicated with dashed lines).

Plant height is polygenically controlled in many cereal crop species (Peiffer et al. 2014; Li et al. 2015; Yano et al. 2016; Würschum et al. 2017; Cosenza et al. 2023). It is unclear, however, to what extent these height-related QTLs can be explained by the initiation and elongation processes. In the present study, using barley as a model, we showed that initiation (number) and elongation (length) could explain ∼40% and ∼50% of height variation, respectively. Given the tradeoff relationship of initiation and elongation (Fig. 1d), we argue that measuring the overall adult plant entity as the quantitative readout may not be sufficient to recapitulate all of the developmental consequences. Affirming this argument is the fact that the *PPD-H1* gene, found to be negatively associated with plant height (TIL) in the present study and in a previous study in wheat (Börner et al. 1993), is in fact a positive regulator of internode elongation (i.e. proximal culm and rachis). Our trait-specific case studies of *HvELF3* (initiation) and the superlocus (elongation) may introduce additional levels of complexity for height. While it has been widely accepted that plant height is the outcome of increases in GA level, the process of increase can be dynamic. For example, in *Arabidopsis*, GA first acts positively for floral induction, then negatively for inflorescence branching (Yamaguchi et al. 2014); similarly, the GA signaling protein DELLA negatively controls meristem size independent of height (Serrano-Mislata et al. 2017). One legitimate consideration is that GA levels need to be repeatedly reset throughout the continuum of plant development, and the timing of this resetting is maintained by the genetic hierarchy of flowering-time genes. Thus, genetic reshufflings of beneficial mutations, recurring over thousands of years of crop evolution, have enabled dynamic adjustments in GA levels at different developmental phases or in different phytomeric units, thereby balancing the initiation and elongation tradeoffs (Fig. 8).

In conclusion, our results demonstrate that segmented plants have developed the capacity to heterochronically modify the growth of different functional units (phytomers) during their lifecycles. We propose that plant architecture should be recognized as a collection of dynamic phytomeric units instead of a uniform entity, particularly when studying how plants interact with heterogeneous environments in the context of crop evolution.

## Materials and Methods

### Plant Materials, Growth Conditions, and Phenotyping

The 25 wild barley founder parents, together with the 247 wild barley introgression lines, are from previous research (Maurer et al. 2015). Wild barley parents for the selected lines are HID003, HID065, HID294, and HID359, respectively. The 358 six-rowed spring barleys were selected from the Federal *Ex-situ* Gene Bank hosted at the Leibniz Institute of Plant Genetics and Crop Plant Research (IPK) (Milner et al. 2019), which were described elsewhere (Kamal et al. 2022; Huang et al. 2023), and were supposed to carry functional *PPD-H1* allele based on the SNP22 reported previously (Turner et al. 2005). *PPD-H1* sensitive lines (BW281) and S41-IL107 were described previously (Digel et al. 2016).

Phenotyping of the wild barley ILs and the D6S population was conducted under controlled greenhouse conditions (photoperiod: 16 h/8 h, light/dark; temperature: 20°C/16°C, light/dark) during the winter season between 2019 and 2022 at the IPK. To phenotype the full set of the D6S panel (358 lines) in a space-limited glasshouse condition, we split the whole panel into two sequential experiments (exp#1, 128 accessions, started at December 2019; exp#2, 256 accessions, started at December 2020), with 25 accessions overlapped between each. We collected only the last two internode lengths from exp#1, and all culm length data in exp#2. BLUEs and raw phenotypic data of the wild barley ILs and the D6S were given at (supplementary table S1 and S2, Supplementary Material online). Barley grains were germinated in a 96-well planting tray for 2 wk, vernalized at 4°C for 4 weeks, acclimatized at 15 °C for a week, and finally transplanted into 9-cm^2^ square pots until maturity. To estimate the effects of different photoperiods on plant development, barley grains were directly sown in 9-cm^2^ square pots in the growth chambers at three continuous photoperiods (8, 12, and 16 h; temperature: 16°C/12°C, light/dark).

We followed an alpha lattice design to control for possible environmental variabilities in the greenhouse, such as different table edges and air conditioner positions. All phenotypic data were collected from the main culm. four (D6S) or five (wild barley ILs) replicates per genotype were collected. We examined the potential rachis node number (PRN) according to Thirulogachandar and Schnurbusch (2021), which was done under a stereo microscope (AxioVision, SE64 Rel. 4.9.1). Final rachis node number (FRN) was counted at anthesis stage. Length-related traits were directly measured with a ruler. Peduncle diameter was measured with a digital caliper. We estimate trait repeatability according to Kamal et al. (2022). Briefly, we first estimated individual variance components of the genotypes, replicates (across the four tables in the glasshouse), and residuals by fixing a mixed-effect linear model using lme4:


$$y_{ij}=\mu +g_{i}+r_{j}+\varepsilon_{ij}$$

In this model, $y_{ij}$ is the recoded phenotypes of the *i*th genotype in *j*th replicates; *μ* is the common intercept term; $g_{i}$ is the genotypic effect from the *i*th genotype; $r_{j}$ is the effect of the *j*th replicates; and $\varepsilon_{ij}\;$ is the residual term. Trait repeatability ($W^{2}$) was estimated as $W^{2}=\sigma_{g}^{2}/(\sigma_{g}^{2}+\sigma_{\varepsilon }^{2}/n)$ where $\sigma_{g}^{2}$ is the genotypic variance, $\sigma_{\varepsilon }^{2}$ is the residual error variance, and *n* is the number of replicates per genotype. Significance of the variance component estimates was tested by model comparison with the likelihood ratio test (Zhao et al. 2015) (supplementary table S3, Supplementary Material online). All phenotypic analyses were done under R (R-3.6.1).

### Estimation of Culm Internode Length Variables

The expression form of indeterminacy can be described as a continuous growth process without constraints, which would expect the newly initiated phytomer to be a simple carbon copy (e.g. length, diameter, and biomass) of the previous one (Barthélémy and Caraglio 2007). Barley shoot apex growth is indeterminate before the transition to the reproductive stage. Thus, the underlying assumption is that the expected length of each culm internode (${\hat{L}}_{i}$) is linearly increased from the proximal (i=1) to the distal (i=n) ends, which can be expressed as ${\hat{L}}_{i}=a{\hat{L}}_{i-1}+b$. By estimating the degree of deviation (sum of residuals) of the observed length ($L_{i}$) to the fitted expected length (${\hat{L}}_{i}$) (supplementary fig. S3c, Supplementary material online), culm internode length oscillation amplitude (*AMP*) can be estimated as follows:


$$AMP=\frac{\sum_{i=1}^{n}\;|L_{i}-{\hat{L}}_{i}|}{TIL}$$

where *TIL* is the total culm internode length, which was normalized to reduce the effect of plant height (i.e. *AMP* can be independent of height).

Due to a variation of node number among lines, and even within the same line with genetic uniformity, it is not applicable to estimate variations of every internode length. It is also not comparable when considering only the few top/bottom internodes because of the mismatched counterparts caused by node number variation (i.e. second internode is the central one from plants having four nodes, but it can be the distal one from those having eight or nine nodes). However, based on the elongation patterns (Fig. 1b and supplementary fig. S3, Supplementary Material online), it becomes clear that plants' culm elongation follows a trisection rule, regardless of the node number. This trisection rule appears to divide the main culm into three compartments: distal, central, and proximal. To facilitate genetic studies, we therefore estimated the average length from the distal (DIL), central (CIL), and proximal (PIL) internodes by using the moving average strategy (supplementary fig. S5, Supplementary Material online). To determine the relative attribution of each height component (INN, DIL, CIL, and PIL) to total culm length (TIL), we first fit a multiple regression model as described in Fig. 1e and then performed the *R^2^* variance decomposition using the Lindeman, Merenda, and Gold (lmg) relative importance algorithm implemented in the relaimpo R package (Grömping 2007).

### Whole-genome Resequencing, SNP Calling and Population Structure

High-quality genomic DNA was isolated and used for library construction using the Nextera DNA Flex library kit. Library preparation and whole-genome sequencing (WGS) using the Illumina NovaSeq 6,000 device at IPK Gatersleben involved standard protocols from the manufacturer (Illumina, Inc., San Diego, CA, United States). The sequencing adapter sequences and low-quality bases were trimmed using cutadapt (v1.15) (Martin 2011). The trimmed reads were then mapped to the Morex reference genome (V2) using minimap2 (v2.20) (Li 2018). Read alignments were sorted by Novosort (v3.06.05) (http://www.novocraft.com/products/novosort/) and then, BCFtools (v1.15.1) (Li 2011) was used to call SNPs, which resulted in 49,526,992 unfiltered SNPs, with 22,405,297 of them having a minor allele frequency of more than 5%.

For population phylogenetic analysis, we first pruned the SNP matrix (22,405,297) using PLINK (Chang et al. 2015) (parameters: –indep-pairwise 50 5 0.2), with a window size of 50 SNPs and step size of 5 SNPs, which we obtained 850,398 independent SNPs (*r*^2^ < 0.2). Phylogenomic tree was constructed based on the distance matrix calculated by PHYLIP 3.68 (https://evolution.genetics.washington.edu/phylip.html) and visualized under iTOL (Letunic and Bork 2016) (https://itol.embl.de/). PCA was performed with PLINK (Chang et al. 2015) using the whole SNP data.

### *P_ST_*–*F_ST_* Comparison

To investigate whether the degree of the phenotypic differentiation observed between different barley subpopulations could be excluded from the possibility of random genetic drifts, we conducted a *P_ST_*–*F_ST_* comparison (Leinonen et al. 2013). *P_ST_* is a phenotypic analog of *F_ST_*. A *P_ST_* >> *F_ST_* suggests that the observed quantitative trait difference would have exceeded the expectation of the influence due to genetic drift, which is indicative of selection. *P_ST_* is measured as the amount of phenotypic variances explained by genetic group membership: $P_{st}=\sigma_{GB}^{2}/\left(\sigma_{GB}^{2}+\sigma_{GW}^{2}\right)$, where $\sigma_{GB}^{2}$ and $\sigma_{GW}^{2}$ are morphological additive genetic variance components between and within subpopulations, respectively. Because barley is a strictly selfing diploid plant species, which means all polymorphic loci are supposed to be homozygous, there is no need to multiply within-population variance $\sigma_{GW}^{2}$ by 2 (Leinonen et al. 2013). Calculation of *P_ST_* was done using the R package pstat v.1.2 (Da Silva and Da Silva 2018). We performed 1,000 replicates of bootstrap resampling to define the confidence interval of each phenotype. Genome-wide estimates of *F_st_* from the comparisons of different subpopulations were obtained using VCFtools (Danecek et al. 2011) by considering the 22,405,297 bi-allelic SNPs (minor allele frequency of ≥5%).

### Association Mapping

For the genome-wide association study (GWAS) in the wild barley introgression lines, we used a simple linear regression model by considering the family information as a covariate, and a significant cutoff as $P<1\times 10^{-3}$, which was similar to previous research (Prusty et al. 2021). GWAS in the D6S population was done by considering a set of 22,405,297 bi-allelic SNPs. We used a linear mixed model that incorporated pairwise genetic similarities (kinship matrix) as the random effect and additional population structure informed by a principal component analysis (PC1–5) as the fixed effect. We ran the GWAS using the software GEMMA (Zhou and Stephens 2012). The significant cutoff ($P<1\times 10^{-5}$) in the D6S population was set with a Bonferroni correction of 1/*n*, where *n* was the number of pruned SNPs (*n* = 100,591) determined by PLINK (window size, 200; step size, 5; *r*2 = 0.05) according to Ren et al. (2022).

### Haplotype Analysis

LD measurements and visualization for SNPs were done with the R package LDheatmap (Shin et al. 2006). To graphically visualize the genotypes within the superlocus (∼100-Mb), we used a sliding window strategy with a window size of 10-Mb and step size of 1-Mb to compress the huge data. In each window, the tested population was forcibly clustered into two groups (assuming all loci are bi-allelic in a diploid species) using the k-means clustering algorithm in R. Major and minor clusters were represented in orange and gray colors in the graphical genotype. Barley accessions were hierarchically clustered according to the resultant genotype of each window (100 windows in total) with the R function hclust. The clustered accessions were colored according to their subpopulation status based on genome-wide SNPs (supplementary fig. S14a, Supplementary Material online). A median-joining haplotype network analysis of the 7 prime candidates within the superlocus was constructed and visualized using PopART (v1.7) (Leigh and Bryant 2015). For this, SNPs within the genomic regions (from start to stop codons) of each gene from the 100 wild and 200 domesticated barleys reported previously (Jayakodi et al. 2020) were used.

### Candidate Genes Functional Enrichment

To summarize the associated candidate genes, significant SNPs for the 14 traits were binned together based on pairwise linkage disequilibrium (LD) decay using the clumping function in PLINK (Chang et al. 2015 [parameters: –clump-p1 $1\times 10^{-5}$, –clump-p2 $1\times 10^{-4}$, –clump-r2 0.3, –clump-kb 5000, –clump-allow-overlap). Thus, for every SNP (index SNP) with $\;P<1\times 10^{-5}$, pairwise *r^2^* values were calculated for SNPs within the 5-Mb surrounding the index SNP (±2.5 Mb); SNPs with an $r^{2}\geq 0.3$ and having a $P<1\times 10^{-4}$ were clumped into bins. Singleton bins without additional SNPs that fell into the criteria were discarded. We recovered 468 bins ranging from ∼10-kb to ∼10-Mb, with a median of ∼2.6-Mb, and encompassing 2,559 high-confidence genes based on Morex reference v2 (Monat et al. 2019). We used the closest homologs in *Arabidopsis* by considering the highest hit of BLASTP search (*e*-value < 1e−5) against the TAIR10 dataset. Functional enrichment analysis was done using Metascape (https://metascape.org/) (Zhou et al. 2019) with default settings. All candidate genes were summarized in supplementary table S6, Supplementary Material online.

### Flowering-Time Genes

*Arabidopsis* flowering-time genes were extracted from the FLOR-ID database (http://www.phytosystems.ulg.ac.be/florid/) as reported previously (Bouché et al. 2015), which included 306 experimentally validated genes participating in diverse pathways (latest updated: 2015 to 2009-23). We identified the flowering-time gene orthogroups between barley and *Arabidopsis* using OrthoFinder 2.4.1 (Emms and Kelly 2019) with default settings. This allowed us to define a set of 268 postulated non-redundant flowering-time genes in barley. Major known genes in barley, including *HvFT1* (*VRN-H3*), *HvCEN*, *PPD-H1*, *VRN-H2*, *HvELF3*, and *HvCO1* were amongst the list (supplementary table S8, Supplementary Material online), suggesting a high degree of conservation on flowering-time control. Morex reference has a natural deletion at the *VRN-H2* locus, we postulated the *VRN-H2* physical position using the flanking sequences of the breakpoints, which was estimated to be at ∼618.6Mb in chr4H of Morex v2.

To test for whether variations in flowering-time genes were more likely to be associated with phytomer initiation and elongation traits with respect to the genome-wide random subset of SNPs, we used a Lambda (*λ*) analysis (Parvathaneni et al. 2020):


$$\lambda =\frac{99\mathrm{th}\;\mathrm{Percentile}[\text{-}\mathrm{LO}G_{10}(\mathrm{Reduced}\;\mathrm{FDR}\;\mathrm{adjusted}\;p\text{-}\mathrm{values})]}{99\mathrm{th}\;\mathrm{Percentile}[\text{-}\mathrm{LO}G_{10}(\mathrm{Genomewide}\;\mathrm{FDR}\;\mathrm{adjusted}\;p\text{-}\mathrm{values})]}.$$

For this, SNPs within 200 kb of the 268 flowering-time genes were used (*n* = 280,687). New False Discovery Rate (FDR)-adjusted *p-values* were calculated using the Benjamini and Hochberg method (Benjamini and Hochberg 1995), and the adjusted top 1% most significant SNPs (99th percentile) were then used to compare with the genome-wide SNPs. We then repeated the random subset 1,000 times with the same SNP number and estimated the *λ* distributions for each trait.

### Cross-species ELF3 Complentation

The 2298-bp full-length coding sequence of barley *ELF3* was amplified from the complementary DNA of cultivar Barke and the 3542-bp *ELF3* promoter from *Arabidopsis thaliana* Col-0, using the primers listed in supplementary table S9, Supplementary Material online. Fragments were cloned into the GreenGate entry modules pGGA000 and pGGC000, respectively. The 669G to 669W mutation was introduced by site-directed mutagenesis using the double primer method and the primers listed in supplementary table S9, Supplementary Material online. Correct integration of the fragments into entry modules was verified by restriction digestion reactions and the cloned sequences verified by sequencing. Final constructs were assembled into the binary vector pFASTR A-G and independently transformed into the *elf3-1* mutant (Jung et al. 2020) through the floral dipping method. Three independent homozygous transgenic lines for each construct were isolated by phosphinothricin selection. Leaf number and flowering time were assessed by growing plants in a potting substrate under long-photoperiod (16-h light and 8-h dark) with light supplied at 100 μmol m^−2^ s^−1^ by cool white fluorescent bulbs. The air temperature was set to 22 °C or 27 °C. Flowering time was determined by counting the number of rosette and cauline leaves when the first buds in the primary inflorescence became visible.

### Determination of the PPD-H1—*SDW1* Regulatory Axis

To examine the regulation of *SDW1* by PPD-H1, we tested the possibility of gene co-expressions using RNA-seq data from previous studies (Digel et al. 2015; Thiel et al. 2021; Huang et al. 2023). We used Kallisto software (Bray et al. 2016) to estimate the transcript abundances (TPM) of *PPD-H1* (*HORVU.MOREX.r2.2HG0088300.1*) and *SDW1* (*HORVU.MOREX.r2.3HG0256590.1*) using Morex genome annotation V2 (Monat et al. 2019) as a reference. Putative *cis*-regulatory regions of *SDW1* were estimated with Assay for Transposase-Accessible Chromatin sequencing (ATAC-seq) data reported previously (Lu et al. 2019). Data processing, read mapping (to Morex reference V2), and accessible chromatin regions identification were done according to Lu et al. (2019).

We next tested the direct regulation of *SDW1* by PPD-H1 using the dual-luciferase assay in barley protoplasts. We PCR-amplified three potential regulatory regions of *SDW1* from wild-type BW, including the distal region (Dre, ∼1.8-kb, about 50-kb upstream), the promoter region (Pro, ∼2-kb) and the second intron region (Int, ∼1.4-kb), and then cloned them independently into the pGreenII 0800-LUC vector (Hellens et al. 2005) to generate the *pSDW1^Int/Dre/Pro^*-LUC constructs (reporters). Full-length coding regions (1977-bp) of the *PPD-H1* gene from BW281 (functional) or BW (reduced functional) were cloned into the pGreenII 62-SK vector equipped with a 35S promoter (effectors). To isolate barley protoplasts, 7-day-old etiolated seedlings were hand cut into ∼0.5 mm pieces with a razor blade, and digested with a freshly prepared enzyme solution (Macerozyme, Cellulase R10, Duchefa) for 6 h under darkness with 60 rpm shaking (25°C). Tissues were washed with 50 mL W5 solution (154 mM NaCl, 125 mM CaCl_2_, 5 mM KCl, 2 mM MES [2-(N-morpholino)ethanesulfonic acid] [pH = 5.7]) three times before suspending with an appropriate volume of W5 solution (500 µl per transformation). A 40% Polyethylene Glycol (PEG)-mediated transformation was then applied to deliver the vector combinations into the isolated protoplasts, followed by one more washing step with 880 µl W5 solution, and then resuspended with 1 mL W5. Transformed protoplasts were kept under darkness (25°C) for 16 h before the lysis of the cells. Luciferase and renillia luciferase (REN, for normalization) activities were detected with a Dual-Luciferase Reporter Assay System (Promega, E1910) under the GloMax Discover plate reader system from Promega. Primers used in the dual-LUC assays are given in supplementary table S9, Supplementary Material online.

### RNA *in situ* Hybridization

*SDW1*-specific sequence (402-bp) was PCR-amplified from the total cDNA of BW and cloned into the pGEM-T cloning vector. The resulting plasmid was used as a template for preparing sense (negative control) and antisense probes. A fusion primer set containing a 20-bp T7 promoter sequence (5`-TAATACGACTCACTATAGGG-3`) before the forward primers of sense probes or reversed primer of antisense probes were used. PCR products were then purified and served as templates for in vitro reverse transcription with T7 RNA polymerase. For in situ hybridization, spike samples were fixed overnight with FAA (50% ethanol, 5% acetic acid, and 3.7% formaldehyde) at 4°C, followed by dehydration with ethanol series (50, 70, 85, 95, and 100%) and then embedded with Paraplast Plus (Kendall, Mansfield, MA). A microtome was used to slice the samples (8 µm thick), which were then mounted onto Superfrost plus slides. Tissue pretreatment, hybridization, washing, and coloration were done as described previously (Jackson 1992). Primers for amplifying the probe sequences are given in supplementary table S9, Supplementary Material online.

## Supplementary Material

msae011_Supplementary_Data

## Data Availability

For the raw whole-genome resequencing reads of the D6S panel (358 lines), 168 will be released in conjunction with an upcoming publication; 47 are from a previous publication (Jayakodi et al. 2020); 143 have been submitted to the European Nucleotide Archive database under project id PRJEB61463. The unfiltered VCF variant files of the 358 barley lines have been submitted to the European Variation Archive database under project id PRJEB62782. Genotypic data of the wild barley ILs has been deposited at e! DAL (https://doi.org/10.5447/ipk/2019/20) (Maurer and Pillen 2019).

## Supplementary Material

Supplementary  material is available at *Molecular Biology and Evolution* online.
