# Supplementary material for: Dynamic Phytomeric Growth Contributes to Local Adaptation in Barley
Source: Mol Biol Evol. 2024 Jan 19;41(2):msae011. doi: 10.1093/molbev/msae011 (PMC10837018; doi:10.1093/molbev/msae011)
Supplement: msae011_Supplementary_Data [file msae011_supplementary_data.zip › Supplementary.docx]

**
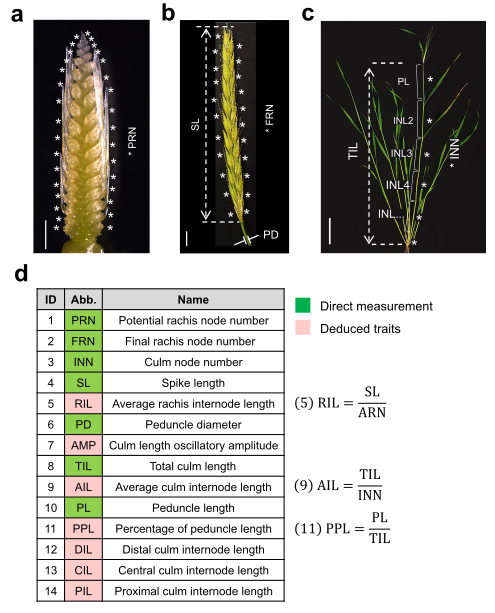
**

**Fig. S1. Summary of the phenotypes.**

**a** – **c**. Representative images showing the phenotyping for node initiation and internode elongation related traits. Reproductive spikes at the maximum yield potential stage (**a**) or anthesis stage (**b**) are used to determine potential rachis node number (PRN) and final rachis node number (FRN), respectively. At anthesis stage, vegetative culms (**c**) are used to collect other phenotypes. Scale bars: 1 mm (**a**), 1 cm (**b**) and 10 cm (**c**). **d**. Trait abbreviations (Abb.) and description of the deduced traits. Other deduced traits (trait id 7, 12 – 14) are summarized in (Supplementary Figs. S3–5).

**
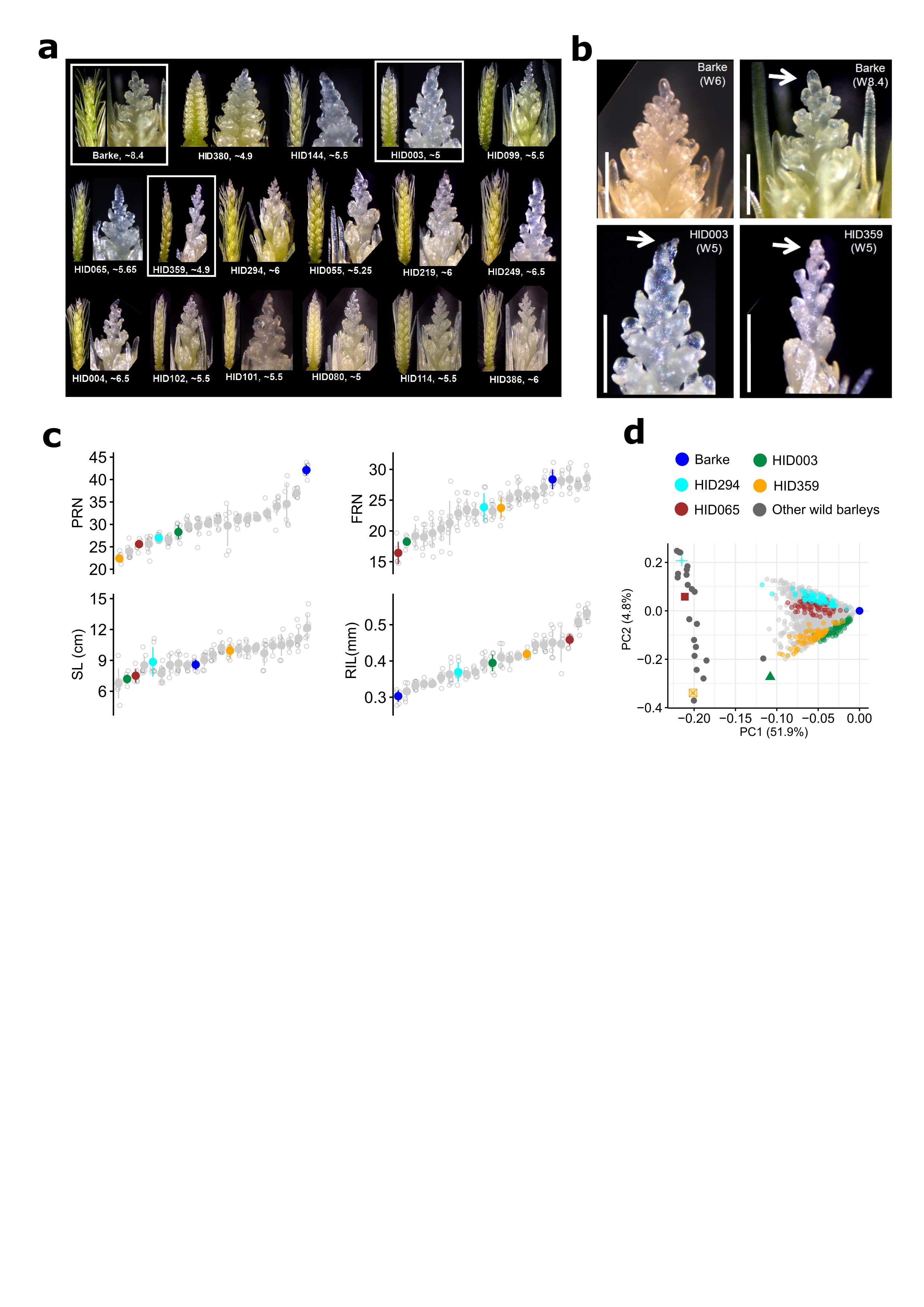
**

**Fig. S2. Spike morphology of the HEB-25 founder parents.**

**a.** Representative spike images from the HEB-25 founder parents. Numbers below each spike represent spike developmental stage defined by Waddington. A close-up view of the tip was included on the right side of each inflorescence. White frames are spikes further highlighted in (**b**). **b**. A further close-up view of the degenerating inflorescences. Note that pre-anthesis tip degeneration (a collapse of the inflorescence meristem) occurred at ~W8.4 in Barke, and at ~W5 in two wild barleys (HID003 & HID359), as pointed out by the arrows. Inflorescence tip of Barke at ~W6 was included for comparison. Scale bars: 500 µm. **c.** Summary of the spike phenotypes from the 25 wild barleys and Barke. Colored genotypes are selected founder parental lines according to (**d**). Light grey colors are the unselected wild barleys. **d**. Genotypic diversity of the four selected sub-populations informed by a PCA plot according to ([Maurer et al. 2015](#_ENREF_55)). Four selected donors and their corresponding HEB lines are colored with non-grey; while the remaining unselected donors and their corresponding HEB lines are colored with dark and light grey, respectively.


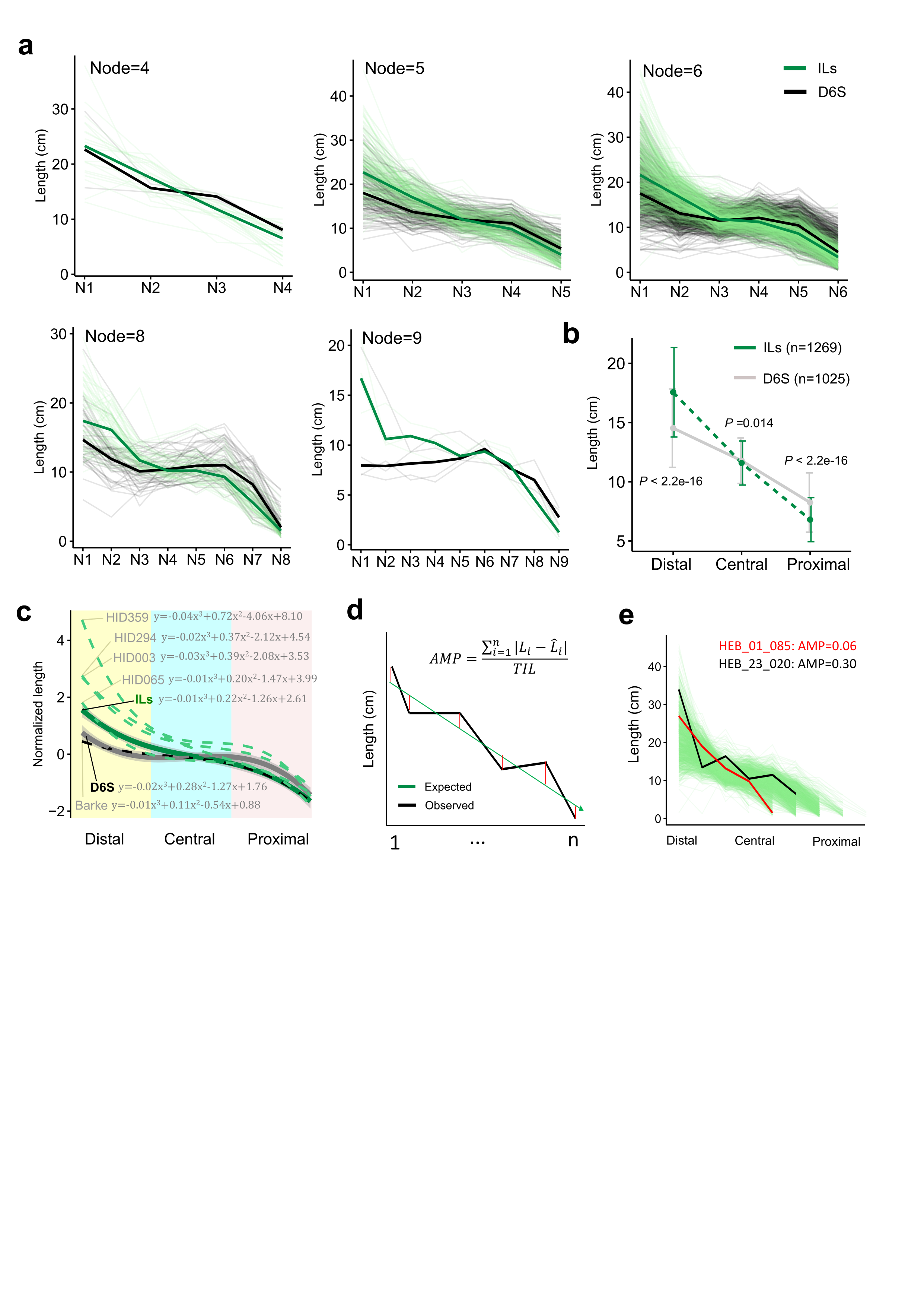


**Fig. S3. Pattern of internode elongation from vegetative culms.**

**a.** Pattern of internode elongation from culms with different node number. See also Fig. 1b. **b**. Average lengths of distal, central and proximal internodes from both populations. **c.** A quartic function modeling the overall patterns of culm internode elongation form the D6S, ILs, as well as the parents. Note that the four wild barley parents overall have both longer distal (DIL) and proximal (PIL) internode length. Compare with wild barley ILs, D6S have shorter DIL, but longer PIL. **d, e**. Estimation of culm oscillatory elongation amplitude (AMP). A graphical depiction of using residuals ($\sigma,$red lines) to estimate AMP. See also Methods. (**e**) illustrates two lines with high (black) or low (red) estimated AMP, in respect to the remaining ILs (green).


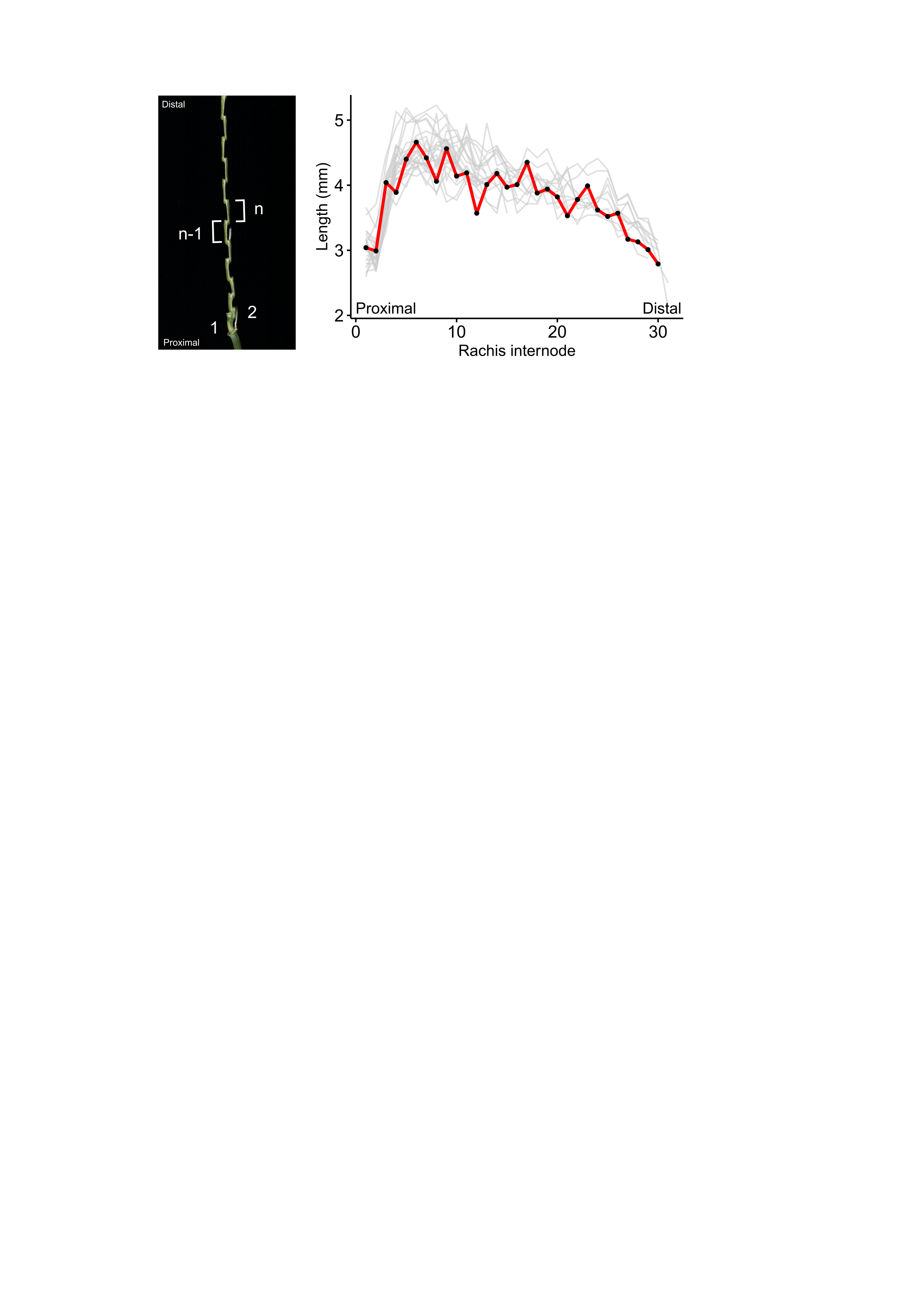


**Fig. S4**. **Pattern of internode elongation from rachis internodes**.

The left side shows the measurement of each rachis internode using an Electronic Vernier Caliper; the right side highlights the rachis internode length from a single representative individual plant (in red color).


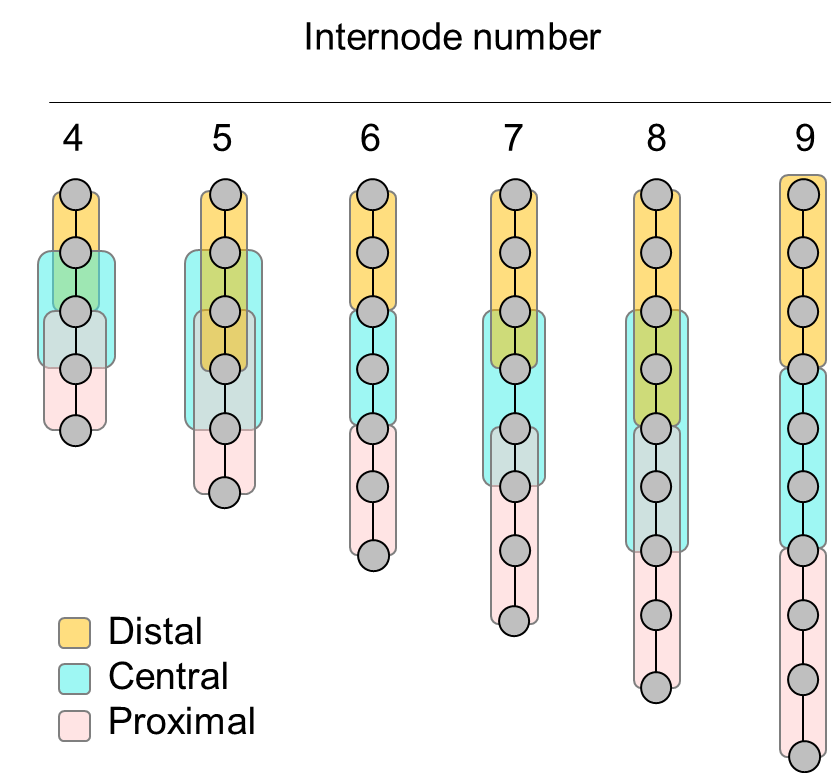


**Fig. S5. Estimation of distal, central and proximal internode length.**

A graphical depiction for the estimation of the average distal, central and proximal internode length by using a moving average strategy. Each grey dot represents a node. Lengths of the internodes within each color boxes are averaged to estimate the average length of distal, central and proximal internodes.


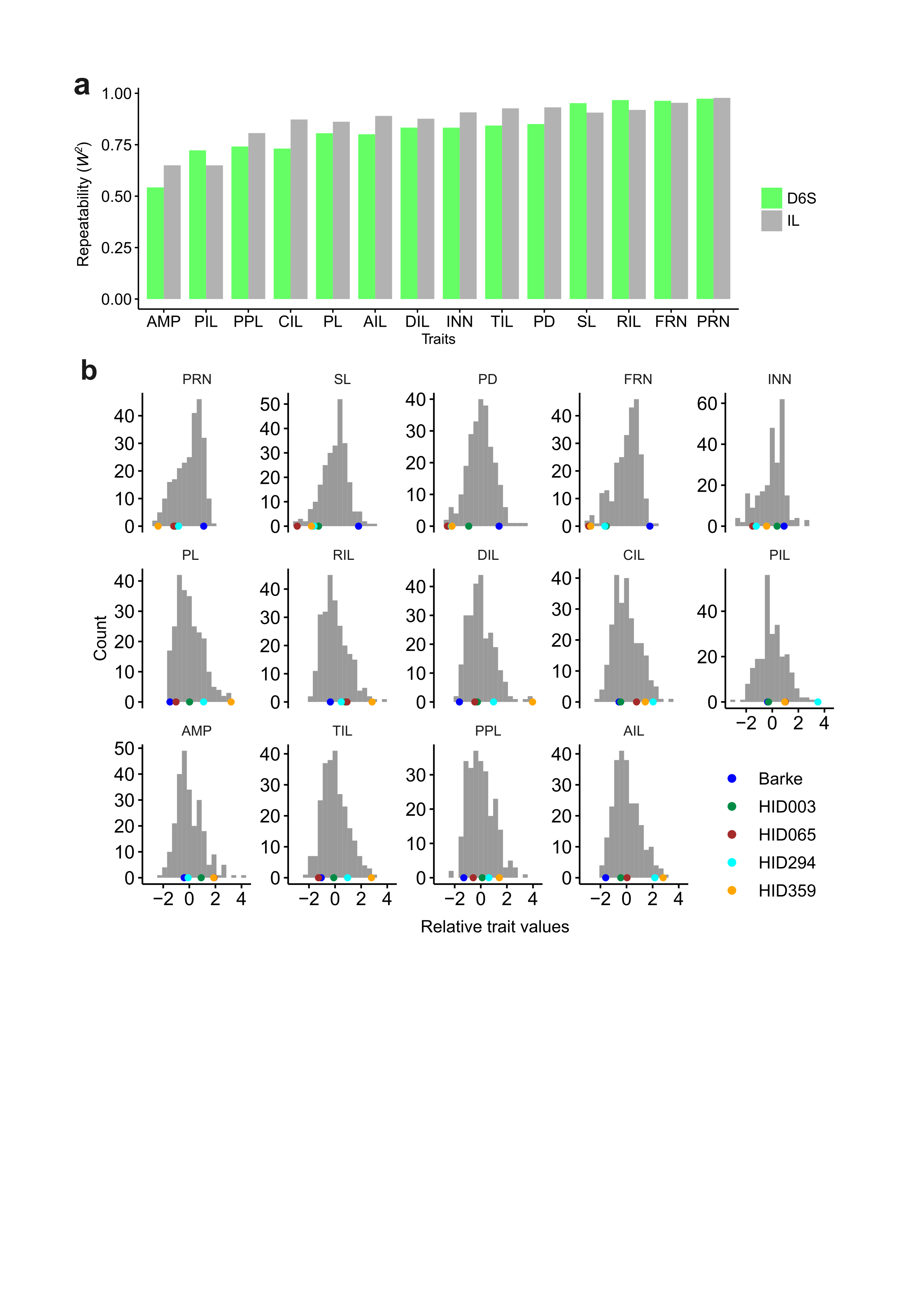


**Fig. S6. Phenotypic variation.**

**a.** Bar plot showing the estimated repeatability (*W^2^*) for the 14 traits in the wild barley ILs (5 replicates) and the D6S population (4 replicates). **b.** Histograms showing the variation of the 14 traits in the wild barley ILs. Parental trait values are highlighted with color dots on the bottom of each histogram. A z-score normalized phenotypic values are shown.

**
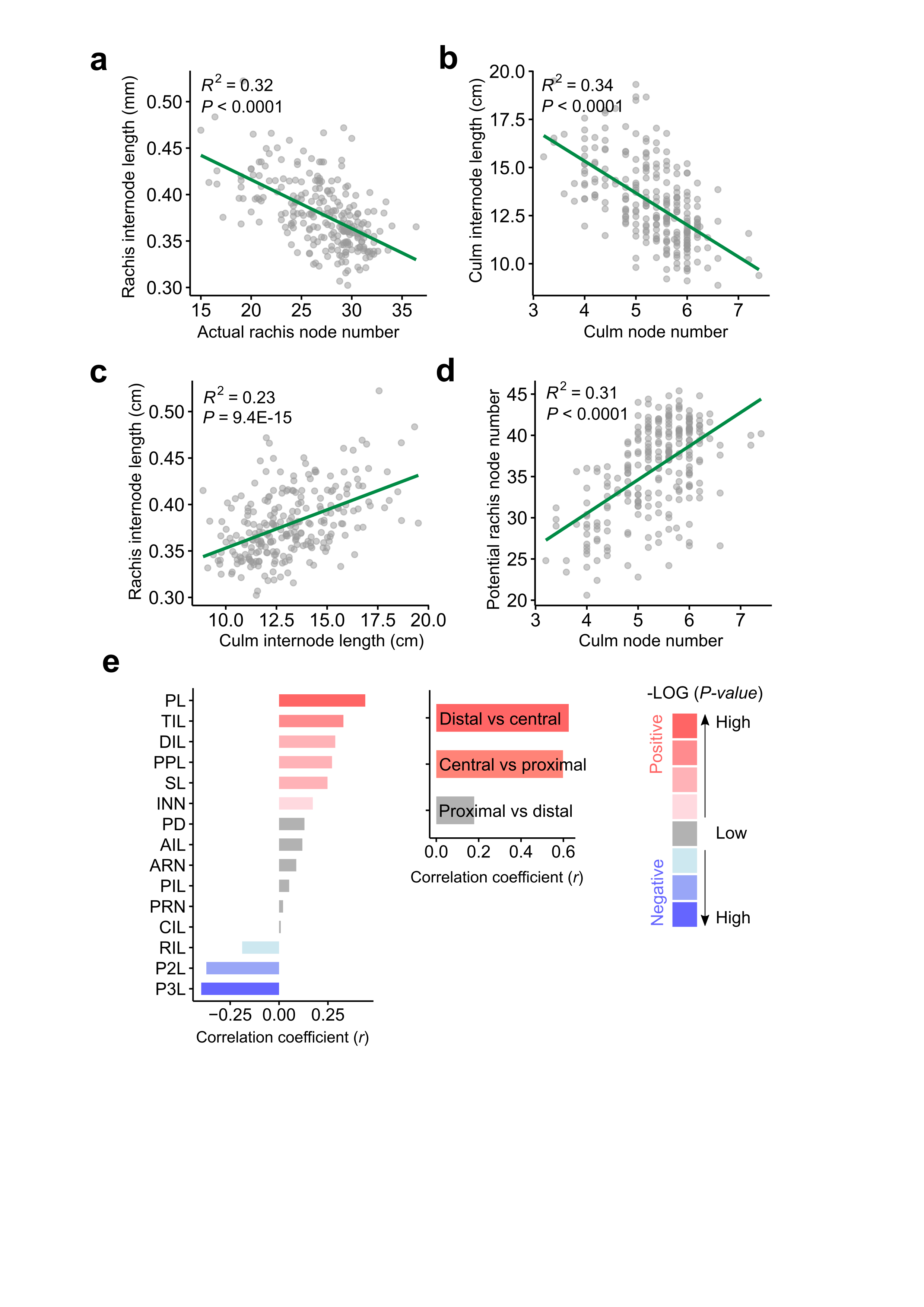
**

**Fig. S7. Phenotypic relationship for node initiation and internode elongation.**

**a – d.** Graphs showing the overall liner relationship from the comparisons of node initiation versus internode elongation (**a**, reproductive; **b** vegetative), or vegetative growth versus reproductive growth (**c**, internode length; **d**, node number). **e.** Relationships of AMP with other phenotypes (left) or among the distal – central – proximal internode length (right) based on the Pearson's correlation coefficient (*r*). Grey color indicates an insignificant correlation between the traits.

**
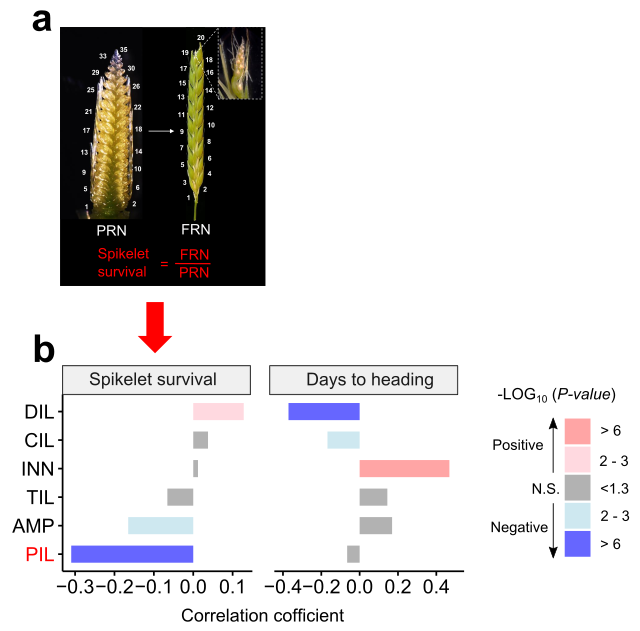
**

**Fig. S8. Correlation analysis of vegetative culm variables with reproductive efficiency**

Left panel depicts the calculation of reproductive efficiency, which is deduced from the fraction of final rachis node number (FRN) by potential rachis node number (PRN). Right panel shows the correlation coefficient of reproductive efficiency with different vegetative culm variables, including distal (DIL), central (CIL) and proximal (PIL) internode length, internode number (INN), total culm length (TIL) and the lengthen amplitude (AMP). Note that among these vegetative culm variables, PIL shows the strongest negative correlation with spikelet survival. Grey color indicates insignificant correlation.


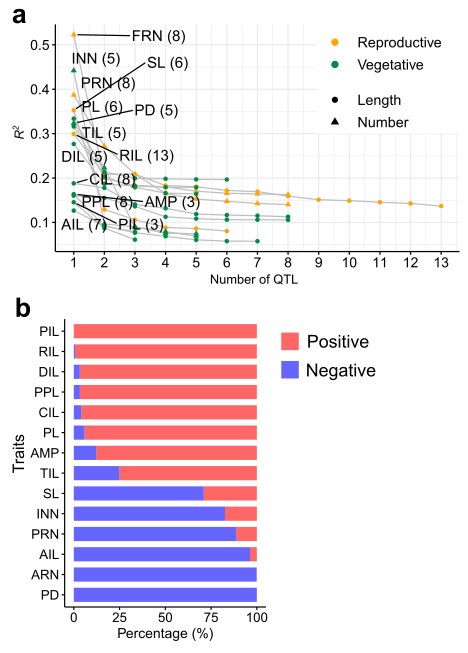


**Fig. S9. Summary of the quantitative trait loci (QTLs) identified in the ILs.**

**a.** Distribution of phenotypic variation explained by each QTL (*R^2^*) and the number of detected QTLs. **b.** Stacked bar graph showing the SNP effects on the phenotypes assayed. Positive or negative represent wild barley alleles will positively or negatively affect the traits under Barke background, respectively.


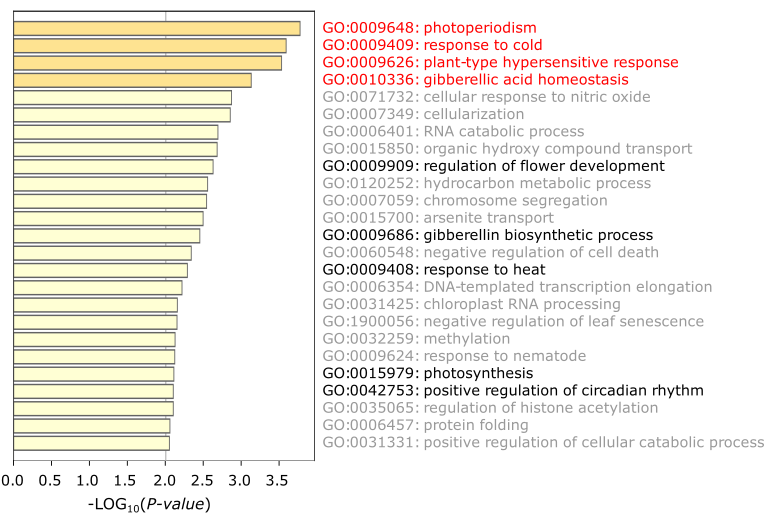


**Fig. S10. GO enrichment analysis of 2,560 GWAS candidates identified in the D6S**

The closest barley homologs of *Arabidopsis* genes were first identified through a BLASTP search. Only the best hit of each gene (e-value<1e-05) was used. Functional enrichment analysis was done with Metascape (https://metascape.org). The top 4 enriched terms are highlighted with red color, other less significantly enriched terms potentially related to adaptation are highlighted with black.


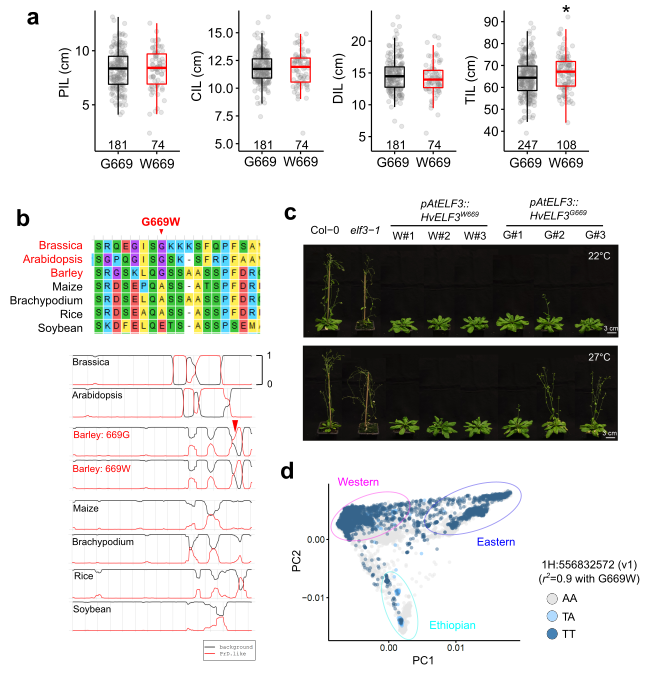


**Fig. S11. HvELF3 G669W variant and the functional consequence.**

**a.** Comparisons of internode elongation and total culm length. Note that only total culm length (TIL) was significantly changed (**P*<0.05) due to the G669W substitution, but not internode elongation traits. **b.** The G669W is predicted to slightly narrow the prion domain (PrD). In silico prediction was done using the Prion-Like Amino Acid Composition (PLAAC) algorithm (http://plaac.wi.mit.edu/). Top panel shows the sequence alignment surrounding the G669W mutation sites in 7 plant species with (red, inhabiting at colder climates) or without (black, inhabiting at warmer climates) the PrD. **c.** Representative image showing the differential complementation of flowering time for the *Arabidopsis elf3-1* mutant with barley HvELF3^669W^ or HvELF3^669G^ variants. **d.** HvELF3 G669W variation is mainly associated with the PC2 axis that separate Ethiopian barleys from the remaining. See also Fig. 4f.

**
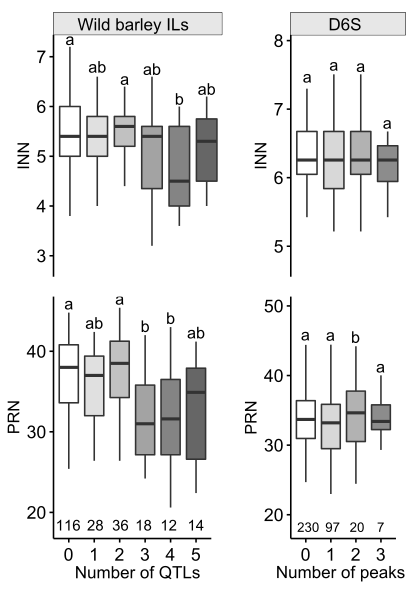
**

**Fig. S12. Effect of the super-locus on phytomer initiation traits.**

Note that similar additive effect for internode elongation was not observed for node initiation (PRN and INN). Letters above boxplot represent statistical significance from one-way analysis of variance (ANOVA) followed by Tukey–Kramer honestly significant difference (HSD) tests.


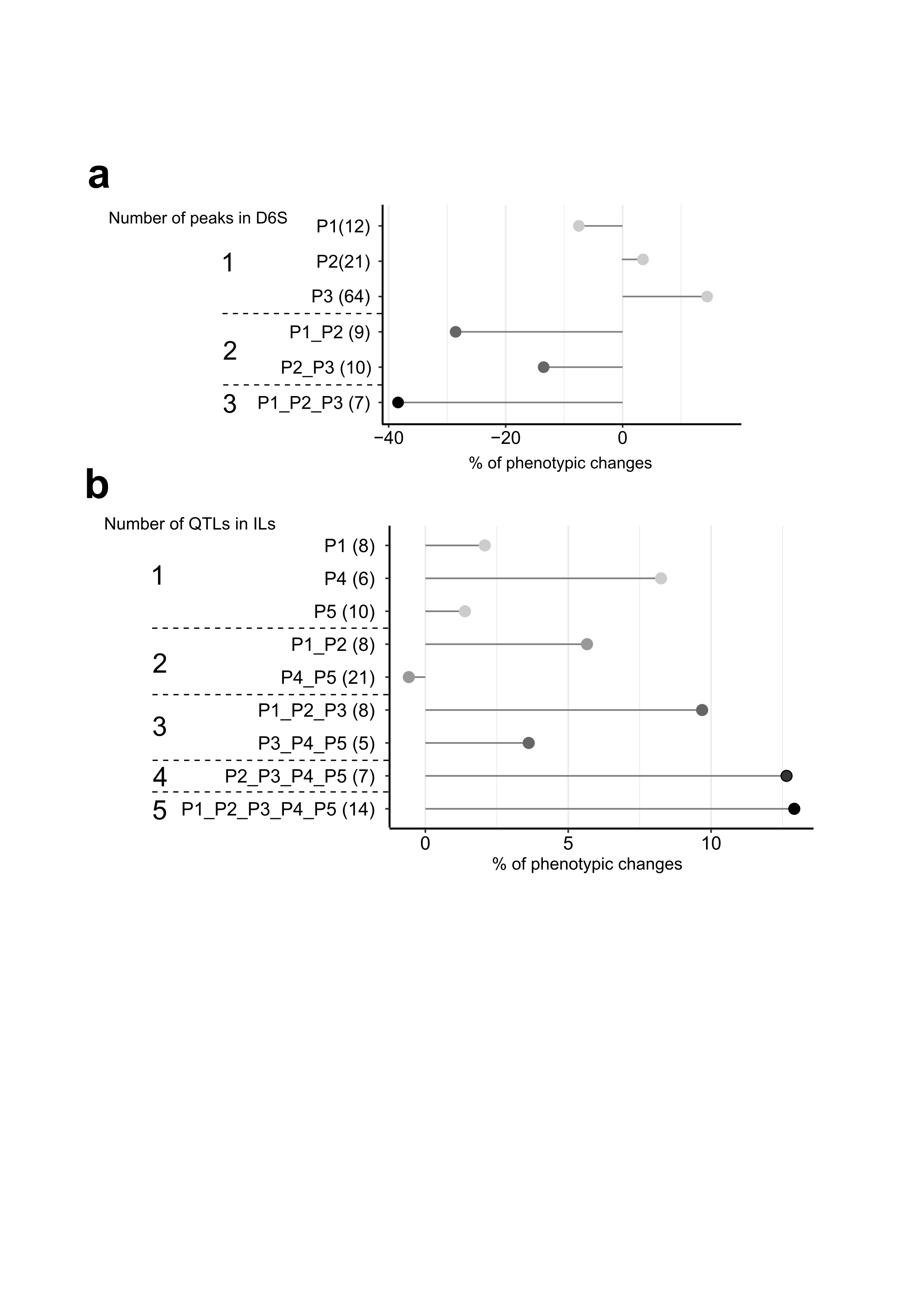


**Fig. S13. Effect of the super-locus on rachis internode length.**

**a**. Allelic combinations within the super-locus in the D6S population. **b**. Allelic combinations within the super-locus in the wild barley ILs. In the D6S population, allelic combinations encompassing P1 could exert stronger phenotypic changes than those without P1 (e.g., P1 vs. P1_P2; P2_P3 vs. P1_P2_P3). In case of the wild barley ILs, P2 seems to be more relevant for achieving phenotypic changes compared with the remaining peaks (e.g., P1_P2 vs. P1; P2_P3_P4_P5 vs. P3_P4_P5). Number in the brackets indicates number of lines from each combination.


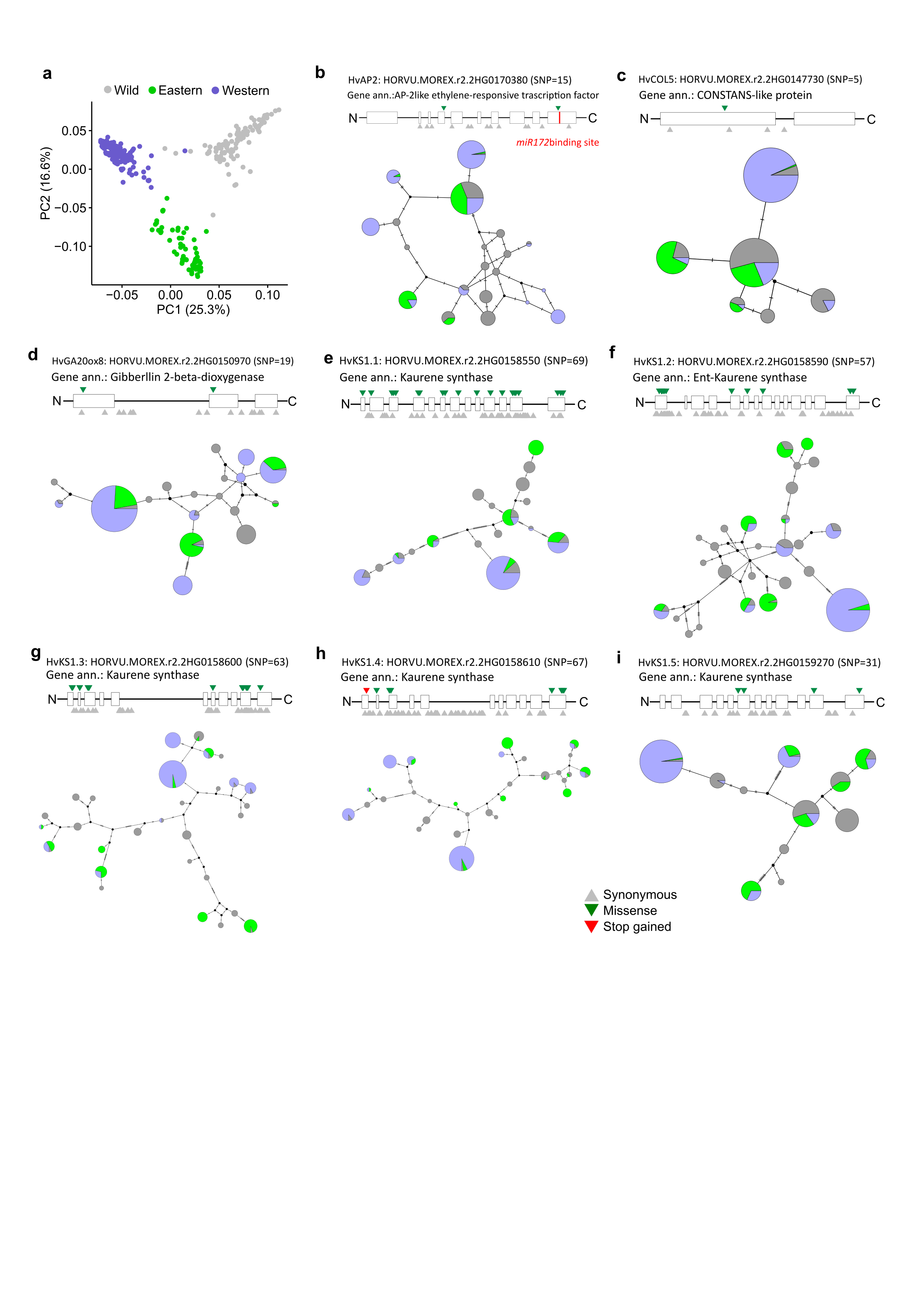


**Fig. S14.**  **Haplotype diversity of the seven prime candidates at the superlocus.**

**a.** Genetic relationship of wild and world-wide domesticated barleys. A PCA based on 31,586,183 genome-wide SNPs reported previously ([Jayakodi et al. 2020](#_ENREF_39)) separates domesticated barleys from the wild barleys, and further defines the domesticated barleys into two clades coincides with Eastern and Western origins. **b – i**. Haplotype diversity of the prime candidates. SNPs from the genomic region (from start codon until stop codon) of each gene were used to construct a median joining network. Gene accession id (Morex V2) and the annotation (ann) are given above each network. Different node colors indicate different clades defined in (**a**). Size of the nodes are proportional to the number of accessions in each node. Gene models (based on Morex v2) are shown above the networks. White boxes represent gene exons. Synonymous SNPs are indicated with grey triangles below each gene model; SNPs that induce amino acid substitutions (non-synonymous) or gain of stop codon are indicated with green or red triangles above each gene model.


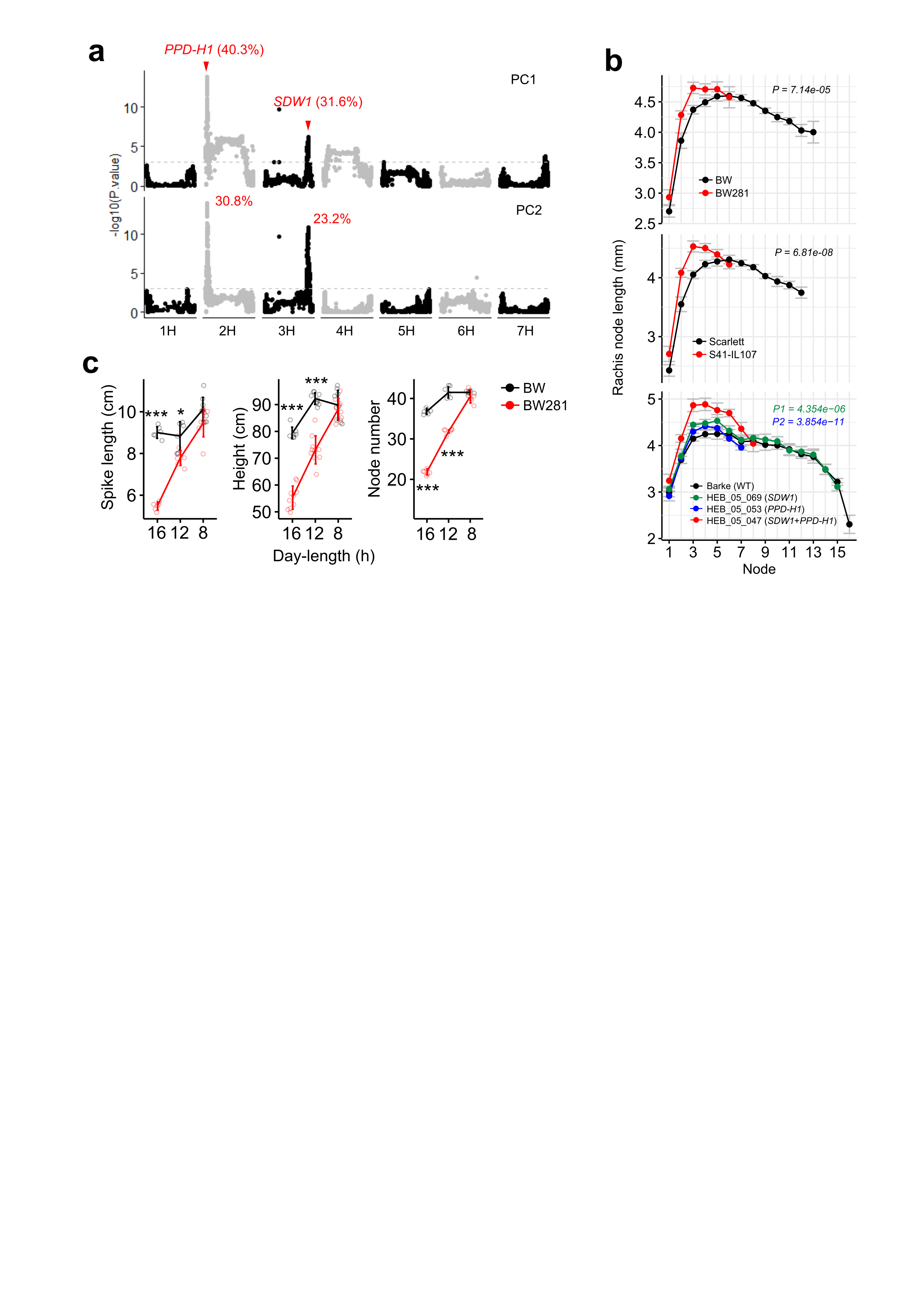


**Fig. S15. Effects of wild barley alleles at the *PPD-H1* and *SDW1* loci**.

**a.** Manhattan plots showing the associations of the *PPD-H1* and *SDW1* loci for the first (PC1) and second (PC2) PCA loadings based for the 14 traits. Percentage of variants explained by each loci (*R^2^*) are indicated. Grey dashed lines are genome-wide threshold at *P* = 0.001. **b.** *PPD-H1* and *SDW1* positively controls rachis internode length. Measurements (one side only from the rachis) were conducted on two *PPD-H1* isogenic lines and their backgrounds [BW281 vs BW (top); S42-IL107 vs Scarlett (middle)], as well as selected introgression lines from the HEB-25 with different wild barley allele introgressions (bottom). Significant values were determined by ANOVA. P1 is from the comparison between HEB-05-047 and HEB-05-069; P2 is from the comparison between HEB-05-047 and HEB-05-053. **c**. Quantitative comparison of spike length (left), plant height (middle) and potential rachis node number (right) between BW and BW281 under 16, 12 or 8 hours (h) of day-length conditions. Significant levels are determined from two-tailed Student’s *t*-test. **P* < 0.05; ****P* < 0.001. *n* = 5 – 9 replicates.


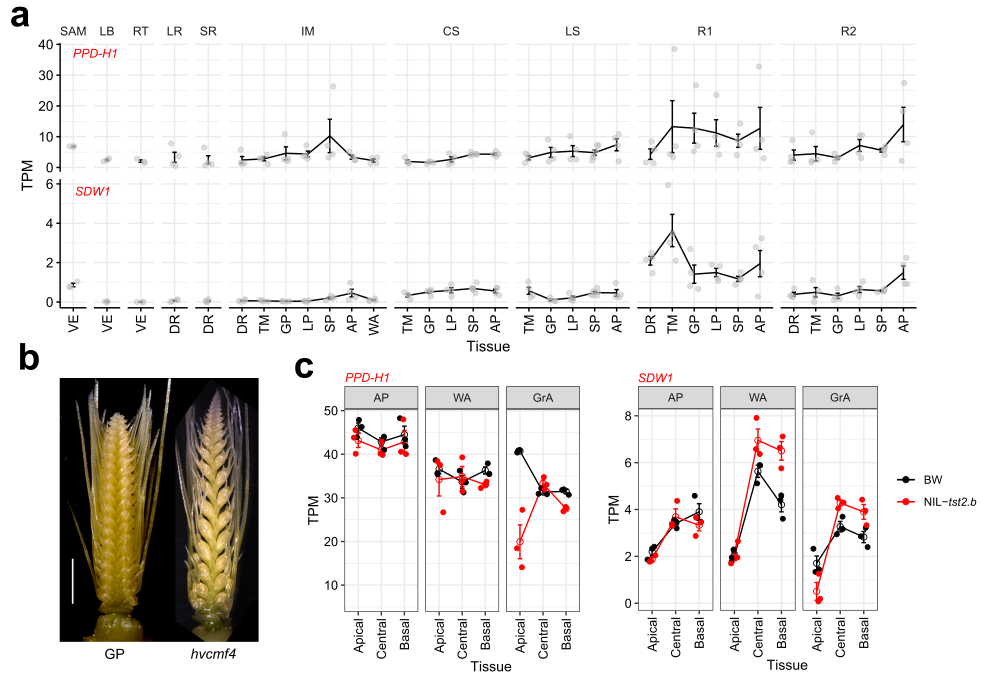


**Fig. S16.** ***SDW1* co-expresses with *PPD-H1* from diverse tissues**.

**a** – **c.** TPM values of *PPD-H1* and *SDW1* from floral meristems (SR, IM, CS, LS, R1 and R2) and non-floral tissues (SAM, LB, RT and LR) in BW (**a**), or different spike sections at three developmental stages in BW and *tst2.b* mutant (Huang *et al.*, 2023) (**b, c**). SAM, shoot apical meristem; LB, leaf blade; RT, root tips; LR and SR, leaf- and spikelet ridges; IM, inflorescence meristem; CS and LS, central and lateral spikelet; R1, rachis; R2, whole spike sections; VE, vegetative stage; DR – WA: spike developmental stages ranging from double ridge (DR), triple-mound (TM), glume primordium (GP), lemma primordium (LP), stamen primordium (SP), awn primordium (AP) and white anther (WA); GrA, green anther stage. GP, Golden Promise. Scale bar: 2mm.


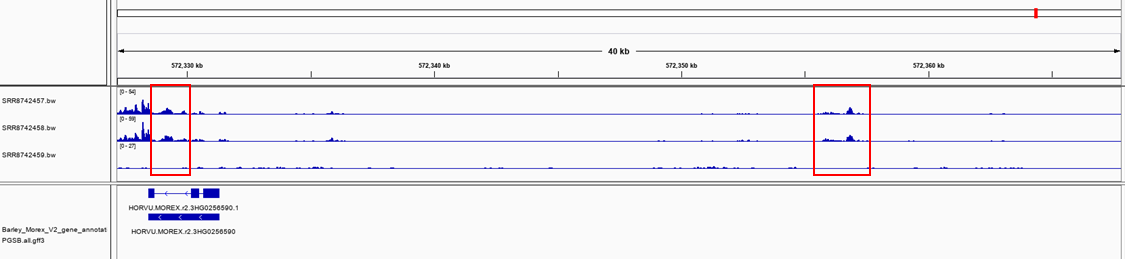


**Fig. S17.**  **Accessible chromatin regions (ACRs) at *SDW1*.**

A snapshot of the Integrated Genomics Viewer browser showing the landscape of ACRs around *SDW1*. ATAC-seq data from ([Lu et al. 2019](#_ENREF_53)) are aligned to Morex reference v2. Identification of ACRs were done according to ([Lu et al. 2019](#_ENREF_53)). Track 1 and 2 are ATAC-seq reads, track 3 is a control. Red frames are two ACRs at *SDW1*, together with the 2-kb promoter regions, are used for dual-LUC assay.


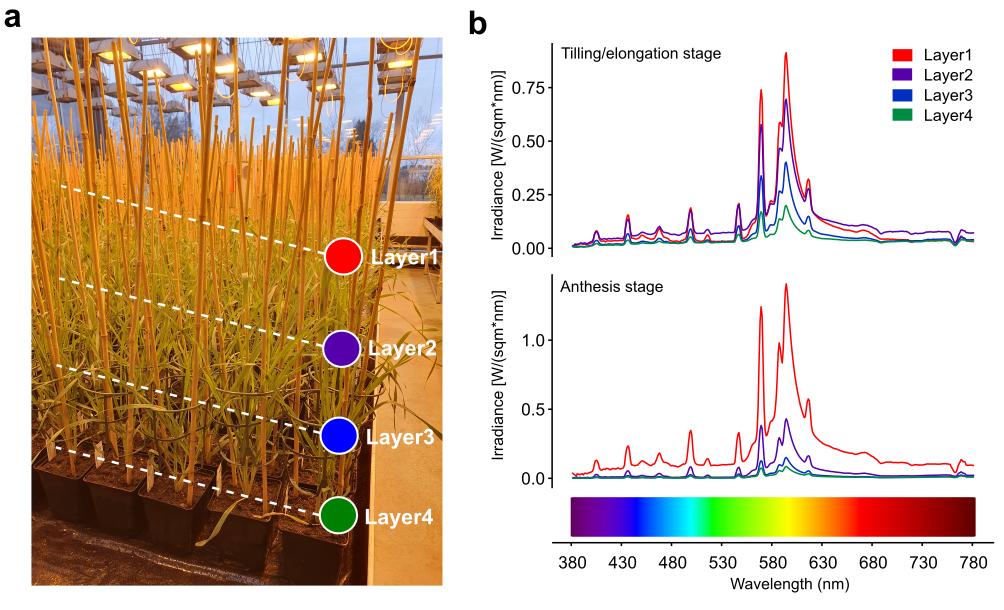


**Fig. S18.**  **Light regimes at different barley canopy layers from the greenhouse.**

**a.** A representative image depicting the different canopy layers at tillering/stem elongation stage. **b.** Light regimes at different barley canopy layers during tillering/stem elongation or anthesis stages. A gradient descent of overall light intensity from Layer1 – Layer4 (distal – proximal) is observed for both stages. Note that light penetrating to the proximal end becomes severely blocked along with growth (tilling – anthesis stage).

**Other supplementary information include:**

**Table S1.** Line information and BLUE values of the phenotypic data

**Table S2.** Raw phenotypic data

**Table S3.** Variance components of genotypes (σ²G), tables (σ²T), and the residuals (σ²error) for the 14 traits in both populations

**Table S4.** Summary of the QTLs detected in the wild barley population

**Table S5.** Summary of the QTLs detected in the D6S population

**Table S6.** Summary of the candidate genes identified in the D6S population

**Table S7.** GO enriched terms of the candidate genes identified in the D6S

**Table S8.** Flowering time genes orthogroups in Arabidopsis and barley

**Table S9.** Primers used in this study
